# TIAM1-RAC1 promote small-cell lung cancer cell survival through antagonizing Nur77-induced BCL2 conformational change

**DOI:** 10.1016/j.celrep.2021.109979

**Published:** 2021-11-09

**Authors:** Aishwarya Payapilly, Ryan Guilbert, Tine Descamps, Gavin White, Peter Magee, Cong Zhou, Alastair Kerr, Kathryn L. Simpson, Fiona Blackhall, Caroline Dive, Angeliki Malliri

**Affiliations:** 1 Cell Signalling Group, Cancer Research UK Manchester Institute, The University of Manchester, Alderley Park SK10 4TG, UK; 2 Cancer Research UK Lung Cancer Centre of Excellence, Manchester, UK; 3 Cancer Research UK Manchester Institute Cancer Biomarker Centre, The University of Manchester, Alderley Park SK10 4TG, UK; 4 The Christie NHS Foundation Trust, Manchester, UK; 5 Division of Cancer Sciences, Faculty of Biology, Medicine and Health, The University of Manchester, Manchester, UK

**Keywords:** TIAM1, RAC, SCLC, neuroendocrine, apoptosis, BCL2, Nur77, small cell lung cancer

## Abstract

Small-cell lung cancer (SCLC), an aggressive neuroendocrine malignancy, has limited treatment options beyond platinum-based chemotherapy, whereafter acquired resistance is rapid and common. By analyzing expression data from SCLC tumors, patient-derived models, and established cell lines, we show that the expression of TIAM1, an activator of the small GTPase RAC1, is associated with a neuroendocrine gene program. TIAM1 depletion or RAC1 inhibition reduces viability and tumorigenicity of SCLC cells by increasing apoptosis associated with conversion of BCL2 from its pro-survival to pro-apoptotic function via BH3 domain exposure. This conversion is dependent upon cytoplasmic translocation of Nur77, an orphan nuclear receptor. TIAM1 interacts with and sequesters Nur77 in SCLC cell nuclei and TIAM1 depletion or RAC1 inhibition promotes Nur77 translocation to the cytoplasm. Mutant TIAM1 with reduced Nur77 binding fails to suppress apoptosis triggered by TIAM1 depletion. In conclusion, TIAM1-RAC1 signaling promotes SCLC cell survival via Nur77 nuclear sequestration.

## Highlights

• TIAM1 is upregulated in neuroendocrine compared to non-neuroendocrine SCLC cells
• TIAM1-RAC1 inhibition decreases SCLC cell viability by increasing apoptosis
• TIAM1 interacts with and maintains Nur77 nuclear localization
• TIAM1-RAC1 inhibition causes Nur77 relocalization and pro-apoptotic BCL2 conversion

## Graphical abstract

**Figure:**
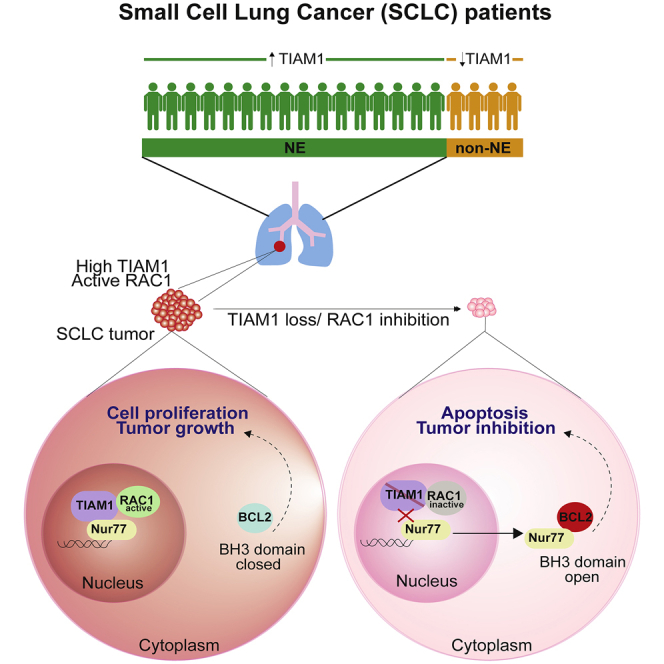

## Teaser

Payapilly, Guilbert et al. identify TIAM1, a selective activator of the small GTPase RAC1, as a gene product upregulated in neuroendocrine SCLC cells. They show that depletion of TIAM1 or RAC1 inhibition leads to SCLC cell apoptosis through cytoplasmic translocation of the orphan nuclear receptor Nur77 and pro-apoptotic BCL2 conformational change.

## Introduction

Small-cell lung cancer (SCLC), an incurable, highly aggressive neuroendocrine (NE) malignancy, is the 6^th^-highest cause of cancer deaths worldwide. Most SCLC patients present with extensive-stage (ES) disease characterized by metastases (Gazdar et al., 2017). 5-year survival for ES SCLC is approximately 2%, unchanged over the past 30 years (Zimmerman et al., 2019). ES-disease patients have few treatment options, receiving platinum-based chemotherapy (with or without radiotherapy) to which they typically and rapidly acquire resistance (Gazdar et al., 2017). Recently, chemotherapy combined with immunotherapy has shown a modest improvement in outcomes for a minority of (molecularly undefined) patients (Horn et al., 2018). Thus, discovery of new therapeutic targets through increased understanding of SCLC biology is urgently required.

SCLC is treated as a homogeneous disease with a marked absence of clinically approved, biomarker-stratified targeted therapies aimed at distinct vulnerabilities. Genome sequencing identified TP53 and RB1 loss-of-function mutations in the vast majority of SCLC cases but failed to identify additional recurrent mutations to classify SCLC subtypes, with the overall genomic landscape characterized by extensive heterogeneity (George et al., 2015; Peifer et al., 2012; Rudin et al., 2012). Distinct “classical” and “variant” morphologies have been described by histology (Carney et al., 1985; de Leij et al., 1985; Gazdar et al., 1985) and transcriptomic profiling has revealed further phenotypic and functional heterogeneity in SCLC (Calbo et al., 2011; Gay et al., 2021; Lim et al., 2017; Stewart et al., 2020; Udyavar et al., 2017; Wooten et al., 2019). An “NE score” based on a 50-gene classifier encompassing differentially expressed NE and non-NE genes classified >80% of SCLC as NE (Zhang et al., 2018). NE SCLC can be further subdivided into SCLC-A and SCLC-N based on expression of the transcription factors ASCL1 and NEUROD1, respectively. Typically, the NE score is lower in SCLC-N than in SCLC-A. Non-NE SCLC has been subdivided into SCLC-Y and SCLC-P based on elevated expression of the transcription factors YAP1, involved in HIPPO signaling, and POU2F3, which signifies tuft cell rather than pulmonary NE cell origin, respectively (Rudin et al., 2019 and references therein).

Intra-tumoral phenotypic heterogeneity is emerging as an important SCLC variable, especially for treatment resistance (Stewart et al., 2020). NE SCLCs can co-express ASCL1 and NEUROD1 (e.g., Simpson et al., 2020). Moreover, SCLC tumors comprise both NE and non-NE cells in varying fractions (Ireland et al., 2020; Simpson et al., 2020). Both cell types cooperate to promote metastasis in a mouse SCLC model (Calbo et al., 2011) and non-NE cells provide NE cells with trophic support and are more chemoresistant (Lim et al., 2017).

Our evolving understanding of inter- and intra-tumoral SCLC heterogeneity is guiding treatment toward an era of precision medicine (Frese et al., 2021). A small subset of SCLC displays MYC amplification or overexpression linked to NE cell transition to non-NE (Ireland et al., 2020; Mollaoglu et al., 2017). MYC amplification/overexpression sensitizes preclinical SCLC models to Aurora kinase inhibitors as well as arginine deprivation (Cardnell et al., 2017; Chalishazar et al., 2019; Mollaoglu et al., 2017). Recently, it was shown that SCLC lacking ASCL1, NEUROD1, and POU2F3 (some ∼18%) is enriched for inflammation and immune response genes and shows improved immune checkpoint blockade response (Gay et al., 2021). Regarding NE tumors, the SCLC-A subtype overexpressed Delta-like protein 3 (DLL3), a NOTCH signaling inhibitor (Saunders et al., 2015), and NOTCH activation in the SCLC-A subtype decreased tumorigenesis (George et al., 2015; Lim et al., 2017). Consequently, recent clinical trials investigated the efficacy of Rova-T, a DLL3-targeted antibody-drug conjugate, as an SCLC therapeutic (Rudin et al., 2017; Wang et al., 2019). However, MYC overexpression cooperates with NOTCH signaling to drive NE transition to non-NE (Ireland et al., 2020), indicating that phenotypic plasticity could undermine monotherapy targeting NOTCH. Thus, the knowledge acquired so far suggests that alternative targets for NE and non-NE SCLC cells are required, as is a combinatorial approach to target both phenotypes.

The small GTPase RAC1 and its guanine nucleotide exchange factor (GEF) TIAM1 promote cell survival, proliferation, migration, and invasion, processes required for tumor growth and metastasis (Maltas et al., 2020; Marei and Malliri, 2017; Porter et al., 2016). TIAM1 depletion increases apoptosis under various conditions (Malliri et al., 2002; Minard et al., 2006; Otsuki et al., 2003; Rygiel et al., 2008). In addition to cancer initiation and progression, TIAM1-RAC1 signaling is involved in neuronal growth and functional maturation (Arimura and Kaibuchi, 2007). A recent study mathematically modeling the factors controlling neuronal stem cell differentiation positioned TIAM1-RAC1 in a positive feedback loop that regulates ASCL1-HES1-mediated neuronal differentiation (Iwasaki et al., 2020). Given these roles of TIAM1-RAC1 signaling, we hypothesized that TIAM1 and RAC1 may be involved in SCLC biology that we herein investigate.

## Results

### TIAM1 and RAC1 expression in SCLC

**Figure 1:**
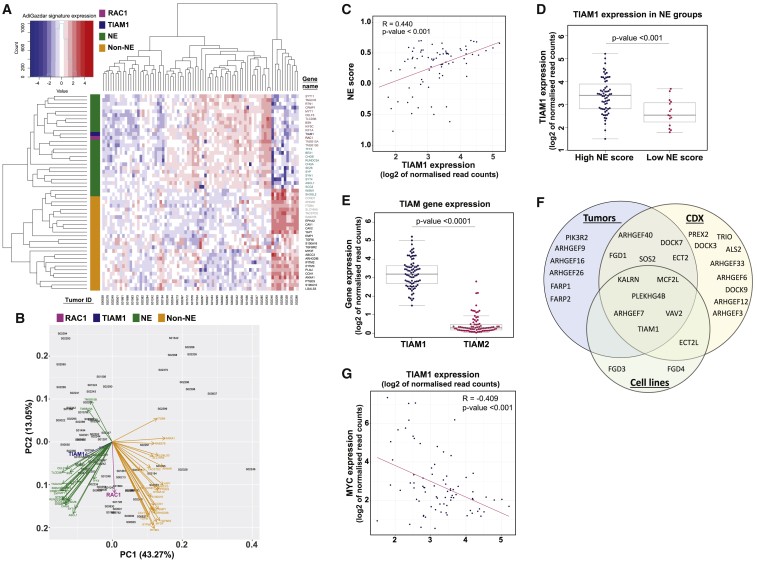
TIAM1-RAC1 signaling is associated with NE SCLC (A) Hierarchical clustering based on NE (green), non-NE (yellow), TIAM1 (blue), and RAC1 (pink) gene expression of SCLC patient tumors represented by a heatmap. (B) PCA plot of the analysis performed in (A). (C) Spearman correlation between 50 gene NE scores calculated for each SCLC patient tumor and TIAM1 gene expression. p < 0.001 (Spearman correlation test). (D) Comparison of TIAM1 gene expression in high-NE- versus low-NE-score SCLC patient tumors. Boxplots represent interquartile range with median TIAM1 gene expression. p < 0.001 (Wilcoxon rank-sum test). (E) Comparison of TIAM1 versus TIAM2 gene expression in SCLC patient tumors. Boxplots represent interquartile range with median gene expression. p < 0.0001 (Wilcoxon rank-sum test). (F) Venn diagrams showing upregulated Rho GEF mRNA (p < 0.05, Wilcoxon rank-sum test) in NE-high patient tumors, cell lines, and CDX models compared to NE-low samples. (G) Spearman correlation between MYC and TIAM1 gene expression in SCLC patient tumors. p < 0.001 (Spearman correlation test). See also Figure S1.

Most, but not all, SCLC tumors and cell lines express genes associated with NE differentiation (Rudin et al., 2019). Gazdar and colleagues described an NE score based on a 50-gene signature (comprising 25 genes overexpressed in NE cells and 25 in non-NE cells) that can be used to classify SCLC tumors/cell lines as either NE (score between 0 and +1) or non-NE (score between 0 and −1) (Zhang et al., 2018). Using this signature, together with TIAM1 and RAC1 expression, we performed hierarchical cluster analysis on RNA-sequencing (RNA-seq) data from 81 primary SCLC tumors (George et al., 2015) (Figure 1A), 44 circulating-tumor-cell-derived explant (CDX) models (Hodgkinson et al., 2014; Simpson et al., 2020) (Figure S1A), and 49 SCLC cell lines (Figure S1F). As expected, SCLC samples clustered into NE or non-NE based on expression of the 25 NE genes (green) and 25 non-NE genes (yellow). Principal-component analysis (PCA) revealed that TIAM1 expression (blue) clustered with NE gene expression (Figures 1B, S1A, and S1F), whereas RAC1 expression (pink) was less consistent. The stronger association of TIAM1 than RAC1 with NE gene expression suggests that in NE SCLC increased expression of select GEFs may lead to increased RAC activity independent of total RAC1 levels, consistent with previous findings in retinoblastoma (Adithi et al., 2006) and nodular melanoma (Dalton et al., 2013).

To assign NE status, NE scores were calculated for each SCLC tumor, CDX model, or cell line. TIAM1 gene expression positively correlated with NE score (Figures 1C, S1B, and S1G) and was higher in SCLC tumors, CDXs, or cell lines classified as NE compared to non-NE (Figures 1D, S1C, and S1H), confirming the association between TIAM1 and NE gene expression. Moreover, we observed that expression of TIAM1 was higher than for its homolog TIAM2 (Figures 1E, S1D, and S1I). Additionally, analysis of all RHO GEF mRNA expression revealed that TIAM1 is the only RAC-specific GEF upregulated consistently in NE SCLC tumors, CDXs, and cell lines (Figure 1F). To further test the NE SCLC association of TIAM1, we looked at the correlation of TIAM1 expression with MYC, as MYC is known to drive transition to non-NE SCLC (Ireland et al., 2020; Mollaoglu et al., 2017). We found that TIAM1 expression negatively correlated with MYC expression in SCLC tumors (Figure 1G) and CDX models (Figure S1E). Taken together, our data demonstrate that TIAM1 expression is associated with NE status in SCLC tumors, CDXs, and cell lines.

### TIAM1 and active RAC1 are required for SCLC cell viability

**Figure 2:**
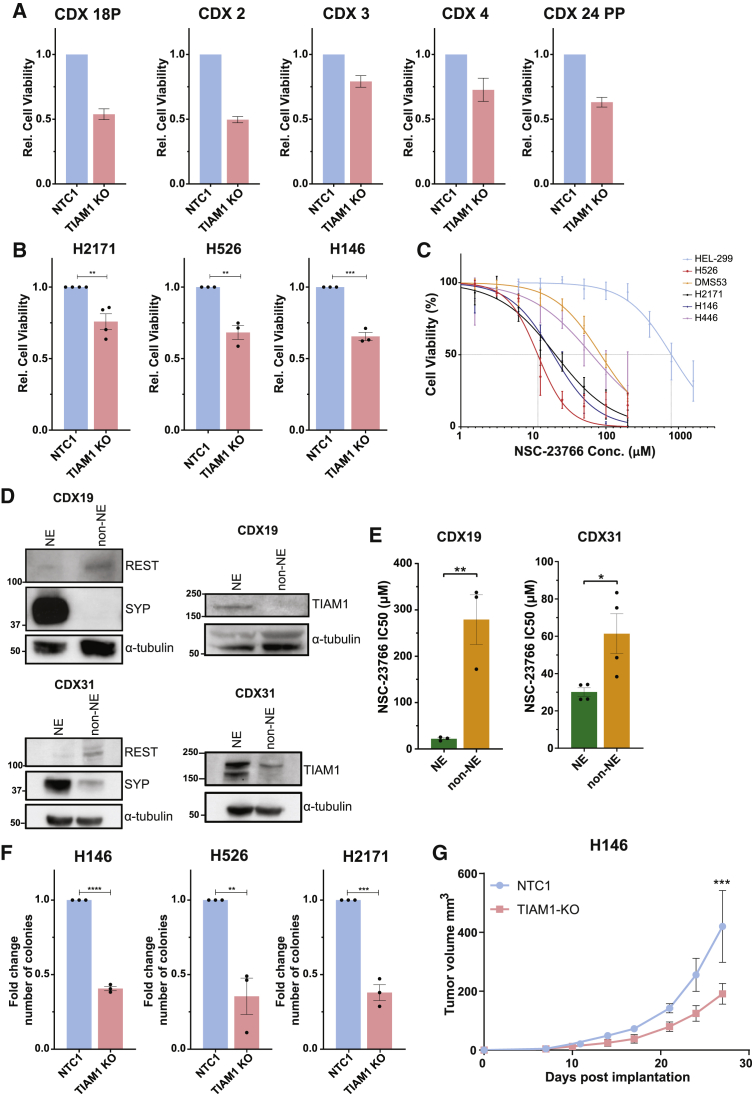
TIAM1 signaling promotes SCLC viability and tumorigenic potential (A) Relative cell viability of TIAM1 KO compared to control (NTC1) CDX2, CDX3, CDX4, CDX18P, and CDX24PP cells. Error bars indicate ±SEM from n = 5 technical repeats. (B) Relative cell viability of TIAM1 KO compared to control (NTC1) H2171, H526, and H146 cells. Error bars indicate ±SEM from n = 3 independent experiments. ^∗∗^p = 0.0043 for H2171, ^∗∗^p = 0.0030 for H526, ^∗∗∗^p = 0.0003 for H146 (unpaired t test, two tailed). (C) NSC-23766 dose-response curves for 5 SCLC cell lines and HEL-299. Error bars indicate ±SEM from n = 3 independent experiments with n = 5 technical measurements for each concentration during each independent experiment. See also Figure S2D. (D) TIAM1, REST, and synaptophysin (SYP) expression in the NE and non-NE fractions of CDX19 and CDX31 transition models. Western blots are representative of n = 3 independent experiments. (E) IC_50_ values for 72-h NSC-23766 treatment of the NE and non-NE fractions of CDX19 and CDX31 transition models. Error bars indicate ±SEM from at least n = 3 independent experiments. ^∗∗^p = 0.0087 for CDX19, ^∗^p = 0.0288 for CDX31 (unpaired t test, two tailed). (F) Fold change in the number of colonies of TIAM1 KO compared to control (NTC1) H146, H526, and H2171 cells. Error bars indicate ±SEM from n = 3 independent experiments. ^∗∗∗∗^p < 0.0001 for H146, ^∗∗^p = 0.0061 for H526, ^∗∗∗^p = 0.0003 for H2171 (unpaired t test, two tailed). (G) Subcutaneous tumor volumes of control (NTC1) or TIAM1 KO H146 cells measured over time. Error bars indicate ±SEM from n = 6 mice. ^∗∗∗^p = 0.0002 (two-way ANOVA, Sidak’s multiple-comparisons test). See also Figure S2.

Given the role of TIAM1-RAC1 signaling in promoting tumor initiation and growth (Maltas et al., 2020) and our above findings that TIAM1 expression positively correlates with NE score, we hypothesized that TIAM1-RAC1 signaling might be important for the viability of NE SCLC cells. We utilized lentivirally delivered CRISPR-Cas9 to knock out TIAM1 in cultures of five CDX models classified as SCLC-A (Simpson et al., 2020); in each case this significantly decreased cell viability (Figure 2A). SCLC CDX cultures are limited to a few passages (Lallo et al., 2019). For further validation, we extended our analysis to NE SCLC cell lines. Lenti-CRISPR-mediated knockout (KO) of TIAM1 in NE SCLC cell lines also resulted in a significant decrease in viable cells in comparison to control cells (Figures 2B and S2A). Additionally, similar results were obtained upon small interfering RNA (siRNA)-mediated knockdown of TIAM1 in 5 NE SCLC cell lines but not in the non-immortalized, non-tumorigenic lung cell line HEL-299 (Figures S2B and S2C).

Because TIAM1 is a RAC-specific GEF (Michiels et al., 1995), we hypothesized that treatment of SCLC cell lines with NSC-23766, a compound that inhibits RAC1 activation by RAC GEFs including TIAM1 (Gao et al., 2004), might also reduce cell viability. Therefore, NSC-23766 sensitivity was tested in SCLC cell lines in comparison to HEL-299 cells. NSC-23766 IC_50_ values were at least 1- to 2-log-fold lower for SCLC cell lines than HEL-299 cells (Figures 2C and S2D). NE and non-NE cell lines grow in culture as suspended clusters or adherent monolayers, respectively (Gazdar et al., 1985). Interestingly, NSC-23766 IC_50_ values for H146, H2171, and H526 clusters (with higher NE scores) were lower than those for the adherent cell lines H446 and DMS53 (with lower NE scores, but nonetheless classified as NE). CDX models 19 and 31 spontaneously generate an adherent cell fraction. Western blotting revealed downregulation of the NE marker synaptophysin in the adherent component versus the suspension component concomitant with upregulation of the non-NE marker REST (Figure 2D), indicating transition of a fraction of cells to a non-NE fate. Interestingly, TIAM1 protein was significantly upregulated in the NE fraction (Figure 2D) coincident with its increased sensitivity to NSC-23766 (Figure 2E). Together, these data show that reducing TIAM1-RAC1 signaling, either through TIAM1 loss or RAC inhibition, decreases viability selectively in NE SCLC cells.

### TIAM1 is required for SCLC cell tumorigenic potential

We next asked whether TIAM1 was required for NE SCLC cell tumorigenic potential by measuring anchorage-independent growth in soft agar and found that H146, H526, and H2171 cells with TIAM1 KO formed significantly fewer colonies than control counterparts (Figure 2F). To validate this *in vivo*, growth was measured for H146-NTC1 and H146-TIAM1 KO tumors following subcutaneous implantation. H146-TIAM1 KO tumors grew slower than H146-NTC1 tumors, with 54.5% tumor growth inhibition at the final time point (Figure 2G). Similar growth inhibition was obtained with H2171-TIAM1 KO tumors (Figure S2E). Because the H146 cells were also engineered to stably express mCherry-luciferase, animals from both H146-NTC1 and H146-TIAM1 KO groups were imaged weekly by bioluminescence (Figure S2F). A week-on-week increase of bioluminescence was observed in both NTC1 and TIAM1 KO tumors, with less bioluminescence in TIAM1 KO tumors compared to time-matched NTC1 tumors (Figure S2G). Thus, we conclude that TIAM1 supports tumor growth *in vivo*.

### Loss of TIAM1 or RAC1 inhibition induces cell death via BAX/BAK-mediated apoptosis

**Figure 3:**
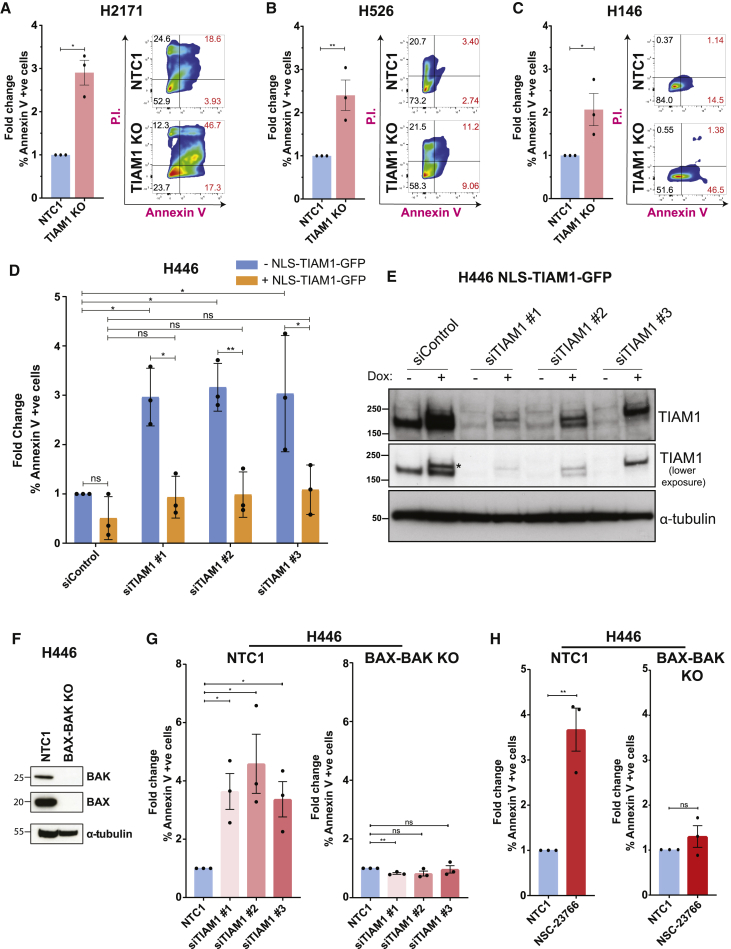
Inhibition of TIAM1-RAC1 induces BAX/BAK-mediated apoptosis in SCLC (A) Fold change in % Annexin V +ve TIAM1 KO cells compared to control (NTC1) H2171 cells with representative fluorescence-activated cell sorting (FACS) plots. Error bars indicate ±SEM from n = 3 independent experiments. ^∗^p = 0.0164 (unpaired t test, two tailed). (B) Same as (A) for H526 cells. ^∗∗^p = 0.0027. (C) Same as (A) for H146 cells. ^∗^p = 0.0463. (D) Fold change in % Annexin V +ve TIAM1 siRNA-treated cells compared to control cells for H446 cells either expressing NLS-TIAM1-GFP (following doxycycline addition) or not. Error bars indicate ±SEM from n = 3 independent experiments. For comparisons within the −dox conditions: ^∗^p = 0.0247 for siControl versus siTIAM1 #1, ^∗^p = 0.0106 for siControl versus siTIAM1 #2, ^∗^p = 0.0184 for siControl versus siTIAM1 #3. For comparisons within the +dox conditions: all comparisons were non-significant (ns). ns for siControl ± dox, ^∗^p = 0.0186 for siTIAM1 #1 ± dox, ^∗∗^p = 0.0100 for siTIAM1 #2 ± dox, ^∗^p = 0.0265 for siTIAM1 #3 ± dox (two-way ANOVA, Sidak’s multiple-comparisons test). (Because the exogenous TIAM1-GFP construct is resistant to only siTIAM1 #3 [see E], GFP-positive cells were gated to analyze only cells where expression of exogenous TIAM1-GFP overwhelmed the effect of the siRNAs.) (E) Representative immunoblot for TIAM1 in control and TIAM1 siRNA-treated cells of (D) (the asterisk indicates NLS-TIAM1-GFP). (F) Immunoblot showing BAX and BAK knockout (KO). (G) Fold change in % Annexin V +ve TIAM1 siRNA-treated cells compared to control siRNA-treated H446-NTC1 and H446-BAX/BAK KO cells. Error bars indicate ±SEM from n = 3 independent experiments. H446-NTC1 cells: ^∗^p = 0.0126 for siTIAM1 #1, ^∗^p = 0.0240 for siTIAM1 #2, ^∗^p = 0.0174 for siTIAM1 #3. H446-BAX/BAK KO cells: ^∗∗^p = 0.00423 for siTIAM1 #1 (all significance tests were unpaired t test, two tailed). (H) Fold change in % Annexin V +ve NSC-23766-treated cells compared to control DMSO-treated H446-NTC1 and H446-BAX/BAK KO cells. Error bars indicate ±SEM from n = 3 independent experiments. H446-NTC1 cells: ^∗∗^p = 0.0049. H446-BAX/BAK KO cells: ns (all significance tests were unpaired t test, two tailed). See also Figure S3.

We next explored the mechanism whereby TIAM1 loss or RAC1 inhibition reduced NE SCLC cell viability. Following 72-h culturing, we detected reduced live TIAM1 KO cells compared to control (NTC1) cells mirrored by a proportional increase in dead cells (Figure S3A), whereas no change in cell-cycle distribution was detected (Figure S3B). Thereafter, NTC1 or TIAM1 KO H2171, H526, and H146 cell lines were stained with Annexin V-APC and propidium iodide (PI). Apoptosis, corresponding to % Annexin-V-positive cells, was measured by flow cytometry. TIAM1 KO resulted in an ∼1.5- to 3-fold increase in apoptotic cells across the 3 cell lines (Figures 3A–3C). Similar results were obtained upon transient downregulation of TIAM1 using two independent siRNAs (Figure S3C). Moreover, apoptosis following TIAM1 knockdown was of comparable magnitude to that observed in cisplatin-treated controls (Figure S3C). In contrast, we observed no increase in apoptosis in the non-tumorigenic cell line HEL-299 upon TIAM1 knockdown; however, some increase in apoptosis was observed upon cisplatin treatment (Figure S3C). As further validation that TIAM1 depletion causes SCLC cell apoptosis, H446 cells were engineered to express doxycycline-inducible exogenous TIAM1 tagged with GFP to enable measurement of apoptosis only in GFP-expressing cells. As expected, TIAM1 knockdown, with 3 independent siRNAs, significantly increased apoptosis compared to control siRNA treatment in H446 cells not expressing exogenous TIAM1 (Figures 3D and 3E). However, expressing exogenous TIAM1 suppressed apoptosis to background levels (Figures 3D and 3E).

Subsequently, we tested whether reduced SCLC cell viability following RAC1 inhibition by NSC-23766 was also due to increased apoptosis. SCLC cell lines were treated with NSC-23766 only, cisplatin only, or both followed by measurement of apoptosis. Similar to TIAM1 knockdown, RAC1 inhibition increased apoptosis comparable to cisplatin treatment in all SCLC cell lines (Figure S3D). In contrast, the non-tumorigenic HEL-299 cell line showed no increase in apoptosis upon treatment with NSC-23766 but did show increased apoptosis following cisplatin treatment (Figure S3D). Interestingly, combining NSC-23766 and cisplatin treatment caused no additive increase in apoptosis (Figure S3D). Taken together, our data show that TIAM1 loss as well as RAC1 inhibition increases apoptosis in SCLC cells.

Apoptosis is mediated by the pro-apoptotic proteins BAK and BAX that oligomerize in the mitochondrial outer membrane, resulting in membrane permeabilization and release of cytochrome *c* that activates cytosolic caspases to induce apoptosis (Green and Reed, 1998). Cells deficient for both BAX and BAK are resistant to apoptotic stimuli (Wei et al., 2001). We created H446 cells lacking BAX and BAK by knocking out both genes with lenti-CRISPR-Cas9 (Figure 3F). The H446-BAX/BAK KO cell line was then used to determine whether cell death induced upon TIAM1 depletion or RAC1 inhibition occurred by BAX- and BAK-mediated apoptosis. TIAM1 knockdown or NSC-23766 treatment increased apoptosis in control H446-NTC1 cells but not in H446-BAX/BAK KO cells (Figures 3G and 3H). The requirement for BAX/BAK indicates that SCLC apoptosis following inhibition of the TIAM1-RAC1 pathway occurs by the intrinsic pathway.

### Pro-apoptotic BCL2 BH3 conformational change upon TIAM1-RAC1 inhibition

To investigate how TIAM1 depletion caused apoptosis in SCLC cells, we first assessed whether levels of pro-survival BCL2 family proteins BCL2, BCLXL, and MCL1 decreased following TIAM1 knockdown. However, no decrease was observed (Figure S4A). SCLC tumors are characterized by deletions or loss-of-function mutations of TP53 (George et al., 2015; Peifer et al., 2012; Rudin et al., 2012) and TP53 inactivation impairs upregulation of BH3-only pro-apoptotic proteins (Villunger et al., 2003). Therefore, an increase in the levels of BH3-only pro-apoptotic proteins was unlikely to explain increased apoptosis following TIAM1 loss.

**Figure 4:**
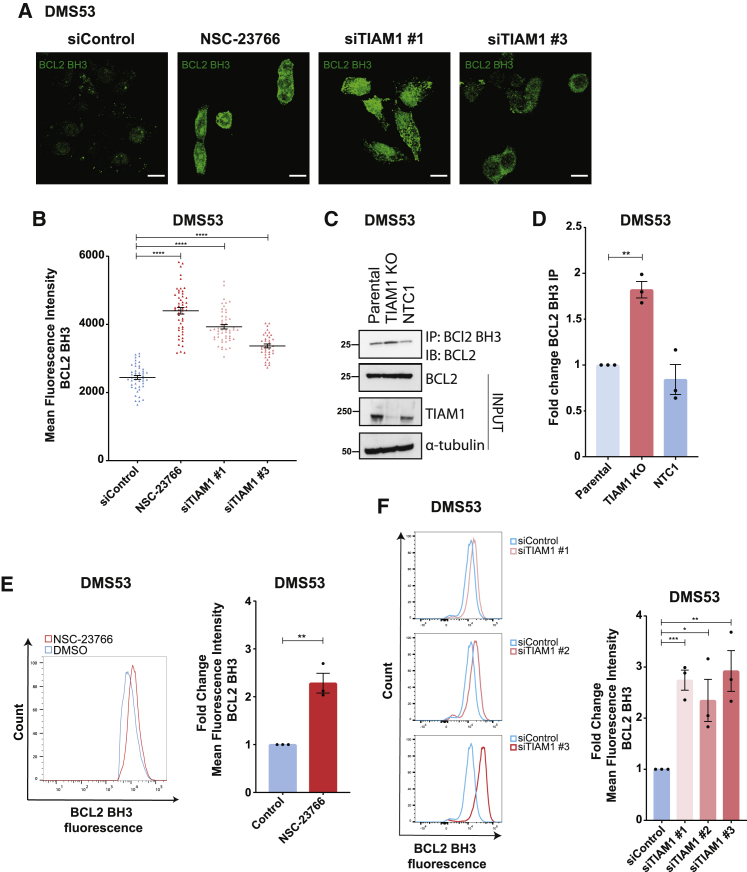
Inhibition of TIAM1-RAC1 induces BCL2 BH3 domain exposure in SCLC cells (A) Representative images of cells stained with the BCL2-BH3-domain-specific antibody in control, NSC-23766-treated, or TIAM1 siRNA-treated cells. Scale bars, 10 μm. (B) Quantification of mean staining intensity of (A). Error bars indicate ±SEM of n > 38 cells for each condition. ^∗∗∗∗^p < 0.0001 (unpaired t test, two tailed). (C) Representative western blot of BH3-domain-exposed BCL2 immunoprecipitated from parental, control (NTC1), or TIAM1 KO DMS53 cells. (D) Quantification of (C). Error bars indicate ±SEM from n = 3 independent experiments. ^∗∗^p = 0.0063 (unpaired t test, two tailed). (E) Representative FACS histograms of BH3-domain-exposed BCL2 fluorescence intensity in DMS53 cells treated with NSC-23776 compared to DMSO and quantification of fold change. Error bars indicate ±SEM from n = 3 independent experiments. ^∗∗^p = 0.0034 for control versus NSC-23766 (unpaired t test, two tailed). (F) Representative FACS histograms of BH3-domain-exposed BCL2 fluorescence intensity in DMS53 cells treated with TIAM1 siRNAs compared to control siRNA and quantification of fold change. Error bars indicate ±SEM from n = 3 independent experiments. ^∗∗∗^p = 0.0009 for siControl versus siTIAM1 #1, ^∗^p = 0.0308 for siControl versus siTIAM1 #2, ^∗∗^p = 0.0085 for siControl versus siTIAM1 #3 (unpaired t test, two tailed). See also Figure S4.

In addition to its well-known pro-survival function, BCL2 can also perform a pro-apoptotic role as first demonstrated for a caspase-cleaved form of BCL2 lacking its N-terminal BH4 domain (Cheng et al., 1997). Moreover, post-translational modifications of BCL2 or interactions of other proteins with its N-terminal loop region (between the BH4 and BH3 domains) cause conformational change resulting in BH3 domain exposure and apoptosis (Deng et al., 2009; Lin et al., 2004). We next examined whether TIAM1 depletion might increase BCL2 BH3 domain exposure by performing immunofluorescence staining with a BCL2-BH3-domain-specific antibody that binds BCL2 upon conformational change (Deng et al., 2009; Lin et al., 2004). We first confirmed that the antibody was BCL2 specific using siRNA to deplete BCL2 (Figures S4B–S4D). Subsequently, we observed increased immunofluorescence signal using this antibody following TIAM1 knockdown or NSC-23766 treatment, indicating BCL2 BH3 domain exposure (Figures 4A and 4B). We corroborated these results by immunoprecipitating more BH3-domain-exposed BCL2 from DMS53-TIAM1 KO cells than control DMS53-NTC1 cells (Figures 4C and 4D), as well as following treatment of DMS53 cells with NSC-23766 (Figures S4E and S4F). We also quantified BCL2 conformational change by flow cytometry and again observed an ∼2-fold increase in BCL2 conformational change in NSC-23766-treated cells or following TIAM1 knockdown (Figures 4E and 4F). Thus, we demonstrated that TIAM1 loss or RAC1 inhibition increases BH3 domain exposure of BCL2, consistent with its pro-death role and the increased apoptosis observed.

### TIAM1 loss promotes Nur77-mediated BCL2 BH3 conformational change and apoptosis

**Figure 5:**
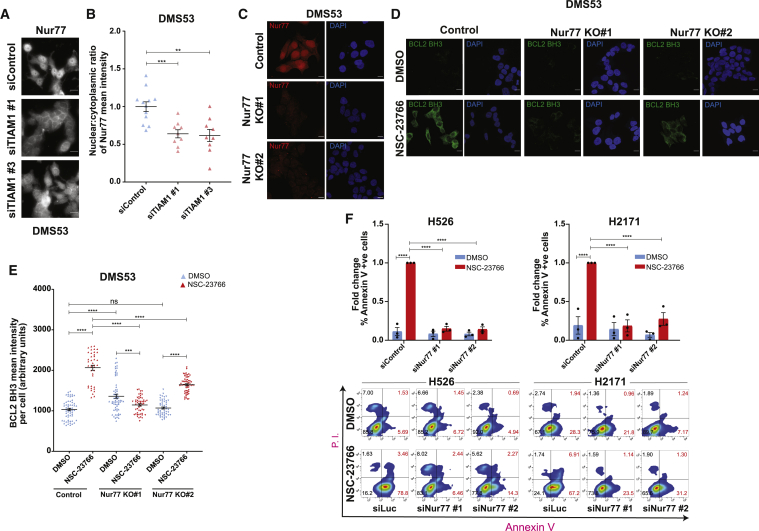
Nur77 mediates BCL2 BH3 domain exposure and apoptosis upon TIAM1-RAC1 inhibition (A) Representative images showing Nur77 localization in siControl or siTIAM1-treated DMS53 cells. Scale bars, 10 μm. (B) Quantification of the nuclear-to-cytoplasmic ratio for (A). Each data point represents one microscopic field of cells. Error bars indicate ±SEM from n = 2 independent biological experiments. ^∗∗∗^p = 0.0006 for siTIAM1 #1, ^∗∗^p = 0.0013 for siTIAM1 #3 (unpaired t test, two tailed). (C) Representative images showing Nur77 staining in control or Nur77 KO DMS53 cells. Scale bars, 10 μm. (D) Representative images showing BCL2 BH3 domain exposure in control and Nur77 KO DMS53 cells (same cells as in C) treated with either DMSO or NSC-23766. Scale bars, 10 μm. (E) Quantification of (D). Error bars indicate ±SEM with n > 40 cells for each condition. ^∗∗∗^p = 0.0002, ^∗∗∗∗^p < 0.0001 (one-way ANOVA, Sidak’s multiple-comparisons test). (F) Fold change in % Annexin V +ve H526 and H2171 cells treated with control or Nur77 siRNAs followed by treatment with either DMSO or NSC-23766 overnight with representative FACS plots and quantification of fold change. Error bars indicate ±SEM from n = 3 independent experiments. For all comparisons, ^∗∗∗∗^p < 0.0001 (two-way ANOVA, Sidak’s multiple-comparisons test). See also Figure S5.

The orphan nuclear receptor Nur77, a typically nuclear protein in non-apoptotic cells, translocates to the cytoplasm and induces apoptosis by interacting with BCL2 and causing BCL2 BH3 domain exposure (Kolluri et al., 2008; Li et al., 2000; Lin et al., 2004; Thompson and Winoto, 2008). Because we observed an increase in BCL2 conformational change upon TIAM1 depletion, we asked whether this was due to a change in Nur77 localization. We observed a decreased Nur77 nuclear:cytoplasmic ratio in TIAM1-depleted cells compared to control cells (Figures 5A and 5B) and in cells treated with NSC-23766 (Figure S5A). We then evaluated whether Nur77 was responsible for the BCL2 conformational change observed following TIAM1-RAC1 inhibition. For this, we created Nur77 KO DMS53 cells (Figure 5C) and assayed them along with control cells for BCL2 conformational change following NSC-23766 treatment. NSC-23766-treated Nur77 KO cells had significantly reduced BCL2 BH3 domain exposure compared to control DMS53 cells (Figures 5D and 5E). Our data therefore show that Nur77 is required to induce BCL2 conformational change following TIAM1-RAC1 inhibition.

To determine whether the Nur77-BCL2 apoptosis pathway mediates the cell death induced by TIAM1-RAC1 inhibition, we investigated the effect of Nur77 depletion on apoptosis resulting from TIAM1-RAC1 inhibition. H526 and H2171 cells were treated with two different siRNAs targeting Nur77, followed by treatment with DMSO or NSC-23766. We observed increased % Annexin-V-positive cells following NSC-23766 treatment in siControl-treated cells but not in Nur77-depleted cells (Figure 5F). Therefore, Nur77 was required for NSC-23766-induced apoptosis in SCLC cells. Furthermore, as shown above, TIAM1 KO increased apoptosis in H526 cells (Figure S5B). However, no increase was observed in TIAM1 KO H526 cells also depleted for Nur77 with two different siRNAs (Figure S5B). Taken together, our results show that TIAM1-RAC1 inhibition causes cell death due to BCL2 BH3 domain exposure mediated by Nur77.

### Molecular characterization of the TIAM1-Nur77 interaction

**Figure 6:**
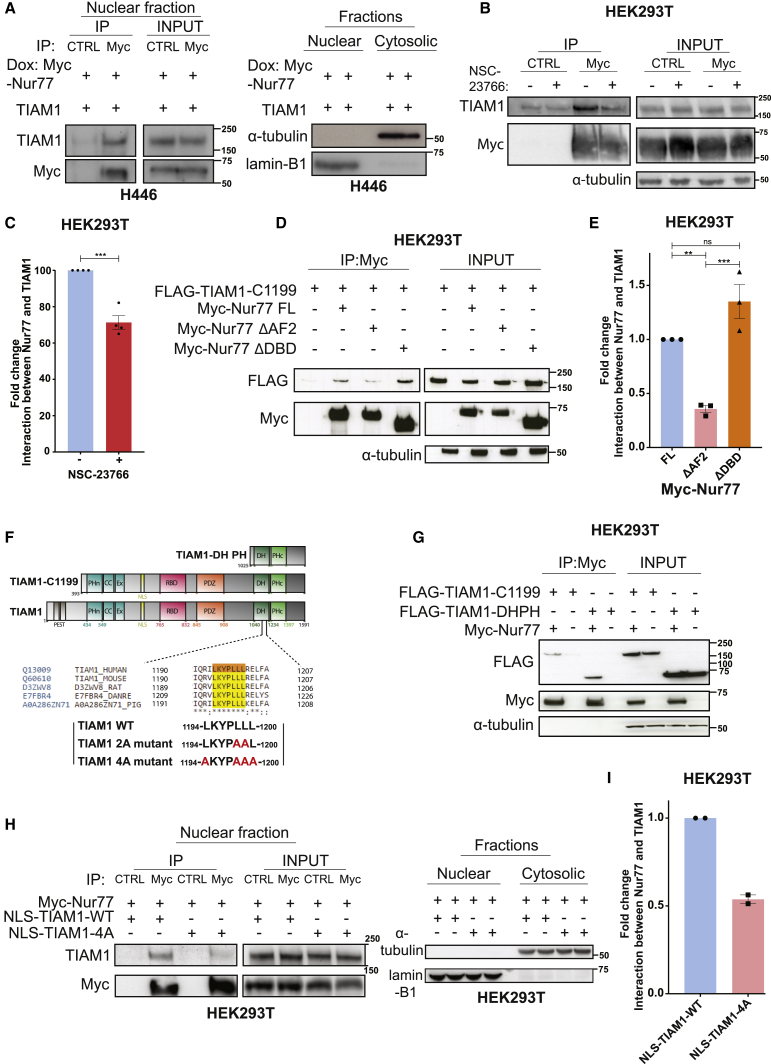
Molecular characterization of the interaction between Nur77 and TIAM1 (A) Exogenous full-length TIAM1 co-immunoprecipitated with exogenous Myc-Nur77 expressed following doxycycline addition from H446 nuclear extracts. Successful isolation of nuclear and cytosolic fractions is also shown. Blots are representative of 3 independent experiments. (B) Representative immunoblot showing the interaction of NLS-TIAM1 with Myc-Nur77 following transfection in HEK293T cells and treatment with either DMSO or 50 μM NSC-23766 overnight and immunoprecipitation with anti-Myc. (C) Quantification of (B). Error bars indicate ±SEM from n = 4 independent experiments. ^∗∗∗^p = 0.0003 (unpaired t test, two tailed). (D) Immunoblot showing the interaction of Myc-tagged full-length or deletion mutants of Nur77 with FLAG-TIAM1-C1199 following their transfection in HEK293T cells and immunoprecipitation with anti-Myc. (E) Quantification of (D). Error bars indicate ±SEM from n = 3 independent experiments. ^∗∗^p = 0.0064, ^∗∗∗^p = 0.0007 (one-way ANOVA, Sidak’s multiple-comparisons test). (F) Schematic of full-length and C1199 and DH-PH mutants of TIAM1. Alignment of the amino acid sequences of TIAM1 containing the LXXXLLL motif is highlighted orange for human and yellow for other species. Leucine-to-alanine substitutions to create the TIAM1 2A or TIAM1 4A mutants are shown in red. (G) Immunoblot showing the interaction of Myc-Nur77 with either FLAG-TIAM1-C1199 or FLAG-TIAM1-DH-PH following transfection in HEK293T cells and immunoprecipitation with anti-Myc. Blots are representative of 3 independent experiments. (H) Immunoprecipitation of NLS-TIAM1-WT and NLS-TIAM1-4A with Myc-Nur77 from nuclear extracts of HEK293T cells. Successful isolation of nuclear and cytosolic fractions is also shown. (I) Quantification of (H) from n = 2 independent experiments. See also Figure S6.

Both TIAM1 and Nur77 localize to the nucleus (Diamantopoulou et al., 2017; Kurdi et al., 2016; Otsuki et al., 2003; Wu and Chen, 2018). Having demonstrated that Nur77 is required for cell death induced by TIAM1-RAC1 inhibition in SCLC cells, we hypothesized that nuclear TIAM1 interacts with Nur77 to promote SCLC cell survival by sequestering Nur77 in the nucleus away from BCL2. Because currently there are no antibodies available that can detect endogenous Nur77 by either western blotting or immunoprecipitation, we generated H446 cells expressing doxycycline-inducible Myc-tagged Nur77. Nuclear fractions were then prepared from cells co-expressing Myc-Nur77 and TIAM1, and Nur77 was immunoprecipitated using a Myc-tagged nanobody that does not bind endogenous Myc. Our results showed that exogenous Nur77 interacts with TIAM1 (Figure 6A), consistent with the above hypothesis.

Although the above finding revealed a way for TIAM1 to directly regulate Nur77 localization and function, it does not address why NSC-23766 treatment caused BCL2 BH3 domain exposure, induction of Nur77 redistribution, and apoptosis. We therefore speculated that RAC1 activation affects TIAM1’s ability to sequester Nur77 in the nucleus. Interestingly, we observed a selective reduction in nuclear TIAM1 in NSC-23766-treated cells (Figures S6A–S6D), suggesting that RAC signaling regulates TIAM1 localization that in turn impacts Nur77 nuclear sequestration. To examine whether RAC signaling could further influence TIAM1-Nur77 interaction independent of effects on subcellular localization, we tested the effect of NSC-23766 treatment on the ability of TIAM1 tagged with a nuclear localization sequence (NLS-TIAM1) to form a complex with Nur77. Interestingly, we observed reduced levels of NLS-TIAM1 co-immunoprecipitating with Nur77 from NSC-23766-treated cells (Figures 6B and 6C). Together, these data indicate multifaceted roles for RAC signaling in regulating the TIAM1-Nur77 interaction.

To identify the Nur77 domain mediating TIAM1 interaction, previously engineered plasmids encoding Myc-tagged Nur77 domain deletions (Figure S6E) (Hu et al., 2017) were expressed in HEK293T cells together with FLAG-tagged N-terminal truncated TIAM1-C1199. Nur77 constructs were then immunoprecipitated with anti-Myc antibody and interaction with TIAM1-C1199 was assessed by western blotting with anti-FLAG antibody. Full-length and DNA-binding-domain (DBD)-deleted Nur77 both interacted with TIAM1-C1199. However, interaction with Nur77 lacking the C-terminal activation function-2 (AF2) domain was reduced (Figures 6D and 6E). Therefore, we conclude that the AF2 region of Nur77 is required for optimal interaction with TIAM1.

AF2 domains in nuclear receptor proteins bind leucine-rich nuclear receptor box motifs LXXLL or LXXXLL (where L = leucine and X = any amino acid) in interacting co-activator and co-repressor proteins (Glass and Rosenfeld, 2000; Hu et al., 2017; Reutens et al., 2001). The TIAM1 peptide sequence contains an LXXXLL motif in its Dbl homology and pleckstrin homology (DH-PH) domain that is conserved across species (Figure 6F). A deletion mutant of TIAM1, containing only the DH-PH domain, was sufficient to interact with Myc-Nur77 (Figure 6G). Previous studies have shown that mutating LXXLL- or LXXXLL-motif leucines to alanines abrogates interaction with AF2 regions of nuclear receptors (Hu et al., 2017). The LXXXLLL motif in the TIAM1 DH-PH domain was mutated to LXXXAAL or AXXXAAA to create a 2A or 4A mutant (Figure 6F). Both 2A (Figures S6F and S6G) and 4A (Figure S6H) TIAM1-C1199 mutants showed decreased Myc-Nur77 interaction compared to wild-type (WT) TIAM1-C1199. Moreover, co-immunoprecipitation assays using nuclear extracts prepared from HEK293T cells expressing WT NLS-TIAM1 (NLS-TIAM1-WT) or NLS-TIAM1 with 4A mutations (NLS-TIAM1-4A) together with Myc-Nur77 showed that NLS-TIAM1-4A had decreased interaction with Myc-Nur77 (Figures 6H and 6I).

Given that the DH-PH domain of TIAM1 is responsible for RAC1 activation, we evaluated the effect of DH-PH domain alanine mutations on RAC1 activation. Pull-down assays indicated that the FLAG-C1199 2A mutant was less capable of activating RAC1 compared to WT FLAG-C1199 TIAM1 (Figures S6I and S6J). Because alanine mutations of TIAM1 decreased its interaction with Nur77 and simultaneously decreased RAC1 activation, it is difficult to separate the requirement for the LXXXLLL motif in mediating a physical interaction with Nur77 from an indirect role through RAC1 activation. We thus conclude that the TIAM1-Nur77 interaction occurs through the AF2 region of Nur77 and the LXXXLLL motif in the DH domain of TIAM1.

### TIAM1-Nur77 interaction is required for SCLC cell survival

**Figure 7:**
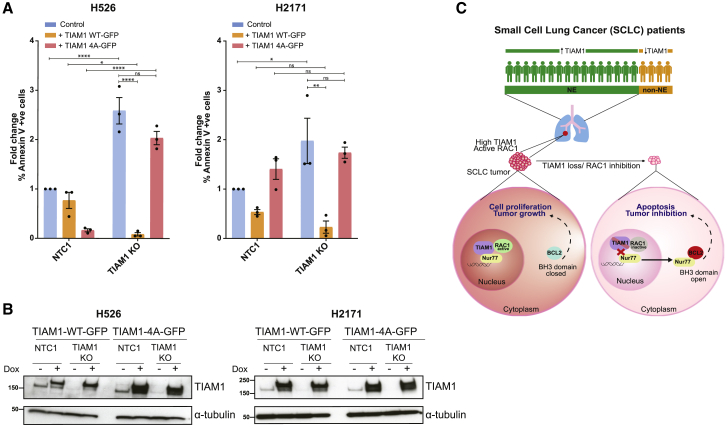
TIAM1-Nur77 interaction is required to prevent apoptosis in SCLC (A) Fold change in % Annexin V +ve control (NTC1) or TIAM1 KO H526 cells (left panel) or H2171 cells (right panel) with or without doxycycline-induced expression of TIAM1 WT-GFP or TIAM1 4A-GFP. Error bars indicate ±SEM from n = 3 independent experiments. For the H526 experiment: ^∗^p = 0.0146 for NTC1 versus TIAM1 KO cells expressing TIAM1 WT-GFP. For all other comparisons: ^∗∗∗∗^p < 0.0001 or ns. For the H2171 experiment: ^∗^p = 0.0244 for NTC1 versus TIAM1 KO in control (−dox) cells, ^∗∗^p = 0.0016 for TIAM1 KO control (−dox) versus TIAM1 KO expressing TIAM1 WT-GFP (all significance tests were two-way ANOVA, Sidak’s multiple-comparisons test). (B) Representative immunoblots of TIAM1 expression in control (NTC1) or TIAM1 KO H526 or H2171 cells with or without expression of TIAM1 WT-GFP or TIAM1 4A-GFP following doxycycline addition (+dox). (C) Model: TIAM1 expression is upregulated in NE SCLC. TIAM1 sequesters Nur77 in the nucleus. Depletion of TIAM1 or inhibition of RAC1 activation by TIAM1 leads to cytoplasmic redistribution of Nur77. In the cytoplasm, Nur77 induces exposure of the BH3 domain of BCL2 promoting apoptosis.

To validate the functional importance of nuclear TIAM1’s interaction with Nur77 in promoting SCLC cell survival, we compared the ability of WT TIAM1 to the 4A mutant to rescue apoptosis after KO of endogenous TIAM1. Control and TIAM1 KO H526 and H2171 cell lines expressing doxycycline-inducible TIAM1 WT-GFP or TIAM1 4A-GFP were created. The presence of the GFP tag enabled measurement of apoptosis only in GFP-positive cells expressing either WT or 4A mutant TIAM1. As expected, TIAM1 KO H526 and H2171 cells showed a significant increase in apoptosis compared to controls (Figure 7A) that was decreased by re-expression of WT TIAM1 but not 4A mutant TIAM1 (Figure 7A). TIAM1 levels before and after inducible expression of the WT or 4A mutant were confirmed by western blot (Figure 7B). These results showed that the LXXXLLL motif in TIAM1 is required for the survival of SCLC cells.

## Discussion

Our study reveals that TIAM1-RAC1 signaling promotes SCLC cell viability. Here we focused on TIAM1’s function in NE SCLC owing to its higher expression in this SCLC type compared to non-NE SCLC, as well as in the NE component of CDX transition models, and that TIAM1 is the only RAC-selective GEF enriched in NE SCLC cells. Moreover, given the current lack of targeted therapies for NE SCLC, which represents ∼80% of all SCLC cases, we were particularly interested in identifying vulnerabilities relevant for this patient population. Additionally, TIAM1 can inhibit the transcriptional activity of MYC (Otsuki et al., 2003). Given the role of MYC in NE-to-non-NE transition (Ireland et al., 2020) and our finding that TIAM1 and MYC expression are inversely correlated, it is possible that increased TIAM1 expression might contribute to sustaining an NE phenotype.

We identify the Nur77-BCL2 apoptosis pathway as the mechanism responsible for cell death induced by TIAM1-RAC1 inhibition in SCLC cells. We show that TIAM1 sequesters Nur77 in the nucleus, which is regulated by RAC activity. Moreover, we infer that abrogating this interaction by TIAM1 knockdown, mutating the LXXXLLL motif, or inhibiting RAC1 activation increases Nur77 cytoplasmic translocation and resulted in BCL2 BH3 domain exposure and apoptosis (for a model, see Figure 7C). Nuclear Nur77 is a known oncogenic survival factor, promoting cell growth and proliferation through transcriptional regulation, whereas cytoplasmic Nur77 is pro-apoptotic and anti-tumorigenic (Kolluri et al., 2003; Lee et al., 2010, 2012). Additionally, cytoplasmic Nur77 is known to induce apoptosis by eliciting BCL2 conformational change (Kolluri et al., 2008; Li et al., 2000; Lin et al., 2004). Further, a short peptide derived from Nur77, which induces apoptosis by BCL2 conformational change (Kolluri et al., 2008), inhibited 3D spheroid growth and caused BCL2-dependent apoptosis in a multidrug-resistant lung cancer cell line (Pearce et al., 2018). However, prior to our study, the identities of endogenous proteins directly implicated in the distribution of Nur77 were unknown. Our findings place TIAM1 upstream of Nur77 and reveal a mechanism of regulating Nur77 localization. How precisely RAC1 promotes the TIAM1-Nur77 interaction is currently unclear and likely multifaceted, and will be the subject of follow-up investigations.

Our study also highlights a further nuclear role for TIAM1, distinct from TEAD regulation that we previously identified in colon cancer cells (Diamantopoulou et al., 2017). Significantly, in T cells, TIAM1 was previously shown to bind to another orphan nuclear receptor, RORγt, that typically interacts with co-activators through its AF2 domain (although the interaction motifs have not yet been defined for TIAM1) and together with RAC1 was required for expression of the RORγt target gene IL17A (Kurdi et al., 2016). Future studies will address possible effects of TIAM1-RAC1 on modulating the transcription factor function of Nur77 as a further potential oncogenic role of TIAM1 in SCLC.

BCL2 is highly expressed in most SCLC tumors (Gazdar et al., 2017). Interrogation of the Broad Institute Cancer Cell Line Encyclopedia (Barretina et al., 2012) indicates that TIAM1 and Nur77 mRNA are also enriched in SCLC compared to other cancer cell lines, suggesting a selective role for this cell survival regulatory pathway in SCLC. Inducing BCL2-BH3-exposure-mediated apoptosis may be a broadly applicable treatment strategy in SCLC with the interaction between TIAM1 and Nur77 being one actionable target. Potentially, a peptide or small-molecule drug that competitively binds to the LXXXLLL motif in the DH-PH domain of TIAM1 or to the AF2 domain of Nur77, disrupting their interaction, could induce Nur77-BCL2-mediated apoptosis in tumors. However, as alluded to above, the LXXXLLL-AF2 interaction mediates numerous nuclear-receptor-co-factor interactions and thus achieving specificity will be necessary. We also showed that NSC-23766 induced Nur77-mediated apoptosis, indicating that inhibiting the activation of RAC1 by TIAM1 could be another potential therapeutic approach. However, this small molecule has an unfavorable pharmacological profile obstructing its use in the clinic. Efforts are ongoing to develop effective alternative TIAM1 and RAC inhibitors (Marei and Malliri, 2017), but efficacy in animal models has not yet been demonstrated.

In summary, we have identified a requirement for the TIAM1-RAC1 signaling module for SCLC survival and highlighted the TIAM1-Nur77 interaction as well as TIAM1-mediated RAC1 activation as potential therapeutic targets.

### Limitations of the study

Although our data implicate both TIAM1 and RAC1 activation in the survival of NE SCLC cells, the precise contribution of RAC1 is unclear. Future studies will address how RAC1 promotes TIAM1 nuclear localization. This could be through either enhancing nuclear import of TIAM1 or suppressing its nuclear export and may involve a RAC1-induced covalent modification of TIAM1. Additionally, it will be interesting to determine whether TIAM1 nuclear localization is regulated by cytoplasmic or nuclear RAC1 or both. We also found that when TIAM1 was forced to enter the nucleus using an exogenous NLS, RAC1 inhibition still impacted the interaction between TIAM1 and Nur77. We speculate that RAC1 activation may indirectly alter TIAM1 or Nur77 covalently with consequences for their interaction. We were unable to create TIAM1 mutants that only affected interaction with Nur77 and not RAC1 activation. This limits our ability to determine whether the effects of TIAM1 depletion on NE SCLC cell survival are mediated entirely through Nur77. We may be able to resolve this issue in the future by expressing exogenous NLS-tagged Nur77 in TIAM1-depleted cells. Additionally, we will address whether the TIAM1-RAC1-Nur77 survival pathway operates in other tumor types.

## STAR★Methods

### Key resources table

**Table:** 

REAGENT or RESOURCE	SOURCE	IDENTIFIER
**Antibodies**		

Sheep polyclonal anti-TIAM1 antibody	R&D Systems	Cat# AF5038; RRID:AB_2303175
Rabbit anti-TIAM1 antibody	Bethyl Laboratories, Inc.	Cat# A300-099A; RRID:AB_2271617
Mouse monoclonal anti-α-Tubulin antibody, Clone DM1A	Sigma-Aldrich	Cat# T6199; RRID:AB_477583
Rabbit polyclonal anti-BAX antibody	Cell Signaling Technology	Cat# 2772; RRID:AB_10695870
Rabbit monoclonal anti-BAK antibody (D4E4)	Cell Signaling Technology	Cat# 12105; RRID:AB_2716685
Rabbit polyclonal Bcl-2 antibody (BH3 Domain Specific)	Abgent/Abcepta	Cat# AP1303a; RRID:AB_2259057
Mouse monoclonal anti-BCL2 antibody (124)	Cell Signaling Technology	Cat# 15071; RRID:AB_2744528
Rabbit monoclonal Bcl-xL antibody (54H6)	Cell Signaling Technology	Cat #2764; RRID:AB_2228008
Rabbit monoclonal Mcl-1 antibody (D35A5)	Cell Signaling Technology	Cat #5453; RRID:AB_10694494
Rabbit polyclonal anti-Nur77 antibody	Proteintech	Cat# 12235-1-AP; RRID:AB_ 10644125
Rabbit monoclonal anti-Lamin B1 (D4Q4Z)	Cell Signaling Technology	Cat# 12586; RRID:AB_2650517
Mouse anti-MYC (9E10)	Santa Cruz	Cat# sc-40; RRID:AB_627268
Mouse anti-FLAG M2	Sigma-Aldrich	Cat# F1804; RRID:AB_262044
Mouse monoclonal anti-RAC1	BDbiosciences	Cat# 610650; RRID:AB_397977
Mouse anti-β-actin (AC-15)	Sigma-Aldrich	Cat# A1978; RRID:AB_476692
Rabbit anti-GFP	Abcam	Cat# ab290; RRID:AB_303395
Anti-Rabbit HRP linked whole Ab	GE Healthcare	NA934; RRID:AB_2722659
Anti-Mouse HRP linked whole Ab	GE Heathcare	NA931; RRID:AB_772210
Donkey anti-sheep HRP conjugated	BIO-RAD	STAR88P; RRID:AB_322719
Donkey anti-rabbit (H+L) Highly cross-absorbed secondary antibody, Alexa Fluor 488	ThermoFischer Scientific UK	Molecular probes A21206; RRID: AB_2535792
Chicken anti-rabbit (H+L) Highly cross-absorbed secondary antibody, Alexa Fluor 647	ThermoFischer Scientific UK	Molecular probes A21208; RRID: AB_141709

**Chemicals, peptides, and recombinant proteins**		

VivoGlo™ Luciferin, *In vivo* grade	Promega	Cat# P1041
NSC-23766	Tocris	Cat# 2161
Cisplatin	Sigma-Aldrich	Cat# 232120
Puromycin	Sigma-Aldrich	Cat# P8833
Blasticidin S HCL	ThermoFischer Scientific UK	Cat# R21001
Geneticin™ selective antibiotic (G418 sulfate)	GIBCO	Cat# 11811031
Doxycycline	Sigma-Aldrich	Cat# D9891
Lipofectamine RNAiMAX Transfection Reagent	ThermoFischer Scientific UK	Cat# 13778075
Fugene® HD Transfection Reagent	Promega Corporation	Cat# E2311
TransIT-LT1 Transfection Reagent	Mirus	Cat# MIR 2300
Lipofectamine 2000 Transfection Reagent	ThermoFischer Scientific UK	Cat# 11668019
Anti-c-Myc Agarose Affinity Gel antibody produced in rabbit	Sigma-Aldrich	Cat# A7470
Myc-Trap Agarose	ChromoTek	yta-20
Binding Control Agarose	ChromoTek	bab-20
Matrigel	Corning	Cat #356237
Strep-Tactin Superflow resin	iba-lifesciences	Cat # 2-1206-025
Biotinylated PAK-CRIB peptide	(Marei et al., 2016)	N/A
DMSO	Sigma-Aldrich	Cat# D8418
GFP-Trap® Agarose	Chromotek	Cat# gta-20
GammaBind™ G Sepharose™	GE Healthcare Life Sciences	Cat# GE17-0885-01
Purified Rabbit IgG Isotype Standard Clone Polyclonal (RUO)	BD PharMingen™	Cat# 550875; RRID: AB_393942
PfuUltra II Fusion HS DNA Polymerase	Aligent Technologies	Cat# 600672
Triton X-100	Sigma-Aldrich	Cat# T9284
NP-40	Sigma-Aldrich	Cat# 492016
Bovine Serum Albumin (BSA)	Roche	Cat# 10 735 078 001
Formaldehyde	ThermoFischer Scientific UK	F/1501/PB08
Prolong™ Diamond Antifade Mountant with DAPI	ThermoFischer Scientific UK	Cat# P36962
NuPAGE™ LDS Sample Buffer (4X)	ThermoFischer Scientific UK	NP0007
NuPAGE™ Sample Reducing Agent (10X)	ThermoFischer Scientific UK	NP0009
NuPAGE™ MOPS SDS Running Buffer (20X)	ThermoFischer Scientific UK	NP0001
NuPAGE™ Tris-Acetate SDS Running Buffer (20X)	ThermoFischer Scientific UK	LA0041
TrypLE ™ Express Enzyme (1X)	ThermoFischer Scientific UK	Cat# 12605036
RPMI 1640 media	GIBCO	Cat# 21875-034
Waymouth’s media	GIBCO	Cat# 31220-023
Heat Inactivated Fetal Bovine Serum (FBS)	Biosera	Cat# FB-1001
10X RPMI solution	Sigma-Aldrich	R1145-500ml
Invitrogen Trypan Blue Stain (0.4%)	ThermoFischer Scientific UK	T10282
Low Melting Lonza SeaPlaque™ Agarose	Lonza	Cat# LZ50101
Indonitrotetrazolium (INT)	Sigma-Aldrich	Cat# I10406
Cell Extraction Buffer	ThermoFischer Scientific UK	Cat# FNN0011
Protease Inhibitor Cocktail	Sigma-Aldrich	Cat# P8340
Phosphatase Inhibitor Cocktail 2	Sigma-Aldrich	Cat# P5726
Phosphatase Inhibitor Cocktail 3	Sigma-Aldrich	Cat# P0044
Insulin	Sigma-Aldrich	Cat# I9278
Transferrin	Sigma-Aldrich	Cat# T3309
β-oestradiol	Sigma-Aldrich	Cat# E8875
Sodium selenite	Sigma-Aldrich	Cat# S5261
Hydrocortisone	Sigma-Aldrich	Cat# H0888
Rock inhibitor Y-27632 2HCL	Selleckchem, Bio Techne	Cat# S1049
StemPro™ Accutase™ Cell Dissociation Reagent	ThermoFischer Scientific UK	Cat# A1110501

**Critical commercial assays**		

CellTiter Glo® Luminescent Cell Viability Assay	Promega	Catt# G7572
Annexin V Apoptosis Detection Kit APC	eBioscience™	Cat# 88-8007
FxCycle PI/RNase Staining Solution	ThermoFischer Scientific UK	F10797
Dead Cell Removal Kit	Miltenyi Biotec	Cat# 130-090-101
MACS columns	Miltenyi Biotec	Cat# 130-042-201
NuPAGE™ 4-12% Bis-Tris Gel 1.0MM 10-well	ThermoFischer Scientific UK	NP0321BOX
NuPAGE™ 3-18% Tris-Acetate Gel 1.0MM 10-well	ThermoFischer Scientific UK	EA0375BOX
Immobilon-P PVDF membrane	Millipore	IPVH00010
X Cell II™ Blot Module	ThermoFischer Scientific UK	EI9051
QuickChange II Site Directed Mutagenesis Kit	Agilent	200521
Q5® Site-Directed Mutagenesis Kit	New England Biolabs	E0554S

**Deposited data**		

SCLC patient tumor RNaseq	(George et al., 2015)	N/A
CCLE SCLC cell line RNaseq	Broad Institute	https://sites.broadinstitute.org/ccle/
CDX RNaseq	ArrayExpress	E-MTAB-8465
Code generated for this study	Zenodo	https://doi.org/10.5281/zenodo.5524543

**Experimental models: Cell lines**		

Human: H2171	Prof. Caroline Dive’s laboratory	Authenticated by MBCF of CRUK MI
Human: H526	Prof. Caroline Dive’s laboratory	Authenticated by MBCF of CRUK MI
Human: H146	Prof. Caroline Dive’s laboratory	Authenticated by MBCF of CRUK MI
Human: H446	ATCC	ATCC® HTB-171™ Authenticated by MBCF of CRUK MI
Human: DMS53	ATCC	ATCC® CRL-2062™ Authenticated by MBCF of CRUK MI
Human: HEL-299	Gift from Dr Michela Garofalo	ATCC® CRL-137™
Human: HEK293T	ECACC (operated by Public Health England)	Cat# 85120602
Human: Lenti-X 293T cells	Prof. Caroline Dive’s laboratory	N/A
H2171 NTC1	This study	N/A
H2171 TIAM1 KO	This study	N/A
H526 NTC1	This study	N/A
H526 TIAM1 KO	This study	N/A
H146 NTC1	This study	N/A
H146 TIAM1 KO	This study	N/A
H446 NTC1	This study	N/A
H446 BAX/BAK KO	This study	N/A
DMS53 NTC1	This study	N/A
DMS53 TIAM1 KO	This study	N/A
DMS53 Nur77 KO#1	This study	N/A
DMS53 Nurr7 KO#2	This study	N/A
H446-iMyc-Nur77	This study	N/A
H446-iNLS-TIAM1 WT-GFP	This study	N/A
H526 NTC1-iTIAM1 WT-GFP	This study	N/A
H526 NTC1-iTIAM1 4A-GFP	This study	N/A
H526 TIAM1 KO-iTIAM1 WT-GFP	This study	N/A
H526 TIAM1 KO-iTIAM1 4A-GFP	This study	N/A
H2171 NTC1-iTIAM1 WT-GFP	This study	N/A
H2171 NTC1-iTIAM1 4A-GFP	This study	N/A
H2171 TIAM1 KO-iTIAM1 WT-GFP	This study	N/A
H2171 TIAM1 KO-iTIAM1 4A-GFP	This study	N/A

**Experimental models: Organisms/strains**		

CDX18P	(Simpson et al., 2020)	N/A
CDX2	(Simpson et al., 2020)	N/A
CDX3	(Simpson et al., 2020)	N/A
CDX4	(Simpson et al., 2020)	N/A
CDX24PP	(Simpson et al., 2020)	N/A

**Oligonucleotides**		

Listed in Table S1	N/A	N/A

**Recombinant DNA**		

pMDLg/pRRE	Prof. Caroline Dive’s laboratory	Addgene plasmid #12251
pRSV-REV	Prof. Caroline Dive’s laboratory	Addgene plasmid #12253
pMD2.G (VSVG)	Prof. Caroline Dive’s laboratory	Addgene plasmid #12259
psVSVG	(Sanjana et al., 2014; Shalem et al., 2014)	Addgene plasmid #8454
psPAX2	(Sanjana et al., 2014; Shalem et al., 2014)	Addgene plasmid #12260
lentiCRISPRv2-puro backbone	(Sanjana et al., 2014; Shalem et al., 2014) Gift from Prof. Stephen Tait	Addgene plasmid #52961
lentiCRISPRv2-puro-NTC1	(Joung et al., 2017) Prof. Caroline Dive’s laboratory	N/A
lentiCRISPRv2-puro-TIAM1 KO	This study (Diamantopoulou et al., 2017; Shalem et al., 2014)	N/A
lentiCRISPRv2-puro-hBAX KO	(Lopez et al., 2016) Gift from Prof. Stephen Tait	Addgene plasmid #129580
lentiCRISPRv2-blast-hBAK KO	(Lopez et al., 2016) Gift from Prof. Stephen Tait	N/A
lentiCRISPRv2-puro-Nur77 KO#1	This study	N/A
lentiCRISPRv2-puro-Nur77 KO #2	This study	N/A
pMIG-Luciferase-IRES-mCherry	(Mallampati et al., 2014)	Addgene plasmid: #75020
pCMV-Myc-Nur77	(Hu et al., 2017) Gift from Prof. Xiao-kun Zhang	N/A
pCMV-Myc-Nur77-del AF2	(Hu et al., 2017) Gift from Prof. Xiao-kun Zhang	N/A
pCMV-Myc-Nur77- del DBD	(Hu et al., 2017) Gift from Prof. Xiao-kun Zhang	N/A
pFLAG-CMV2-TIAM1	(Tolias et al., 2005) Gift from Prof. Kim Tolias	N/A
pFLAG-CMV2-TIAM1 C1199	(Tolias et al., 2005) Gift from Prof. Kim Tolias	N/A
pFLAG-CMV2TIAM1 DH PH	(Tolias et al., 2005) Gift from Prof. Kim Tolias	N/A
pFLAG-CMV2-TIAM1 2A mutant	This study	N/A
pFLAG-CMV2-TIAM1 4A mutant	This study	N/A
pCW57-MCS1-P2A-MCS2 (neo)	Gift from Adam Karpf	Addgene plasmid #89180; RRID:Addgene_89180
pCW57-neo-NLS-TIAM1-GFP	This study	N/A
pCW57-neo-NLS-TIAM1 4A-GFP	This study	N/A
pCW57-neo-TIAM1 WT-GFP	This study	N/A
pCW57-neo-TIAM1 4A-GFP	This study	N/A
pCW57-neo-Myc-Nur77	This study	N/A

**Software and algorithms**		

GraphPad Prism 8.4.3	GraphPad Software Inc	https://www.graphpad.com/scientific-software/prism/
Fiji ImageJ 2.1.0/1.53c	Wayne Rasband (NIH)	https://imagej.net/Fiji
FlowJo 10.6.2	Becton Dickinson & Company (BD)	https://www.flowjo.com/
R version 4.0	R Foundation for Statistical Computing core team	https://www.r-project.org/

### Resource availability

#### Lead contact

Further information and requests for resources and reagents should be directed to and will be fulfilled by the Lead Contact, Angeliki Malliri (angeliki.malliri@cruk.manchester.ac.uk).

#### Materials availability

Plasmids and cell lines generated in this study are available from the lead contact, but we may require a completed materials transfer agreement.

### Experimental model and subject details

#### Mouse xenograft assay

Animal procedures were carried out in accordance with the Home Office Regulations (UK) and the UK Coordinating Committee on Cancer Research guidelines using approved protocols (Home Office Project license no. 70/8386 and Cancer Research UK Manchester Institute Animal Welfare and Ethical Review Advisory Body). Animal studies are reported in compliance with ARRIVE guidelines (Percie du Sert et al., 2020). 1X10ˆ4 disaggregated SCLC cells in 50 μl RPMI media were mixed with 50 μl Matrigel and kept on ice before being injected subcutaneously into the right flank of 6-10-week-old female NSG (NOD/SCID-gamma) mice (purchased from Envigo). Mice were housed in individually vented caging systems in groups of 2-3 mice per cage. Caged mice were kept in an environment maintained under uniform temperature and humidity, under 12 h light and 12 h dark cycles. Mice were monitored by weighing twice weekly. Signs of tumor growth were monitored by palpation and tumor volume (calculated using the formula: 1/2 (length x width^2^)) was measured with calipers twice weekly while total tumor burden did not exceed 1000 mm^3^. Animals were sacrificed according to the Schedule I regulation under the Animals (Scientific Procedure) Act 1986 by neck dislocation followed by confirmation of death by rigor mortis.

#### Cell lines

Cells were cultured on plastic plates or flasks in incubators at 37°C with 5% CO_2_. Parental cell lines were authenticated and routinely checked for mycoplasma contamination by the Molecular Biology Core Facility (MBCF) of the CRUK Manchester Institute. Cell lines generated in this study were engineered using lenti-CRISPR/Cas9 technology and/or lentiviral mediated transduction of inducible gene expression plasmids, as specified. HEK293T, Lenti-X-293 and SCLC cell lines: H526, H2171 and H446 were cultured in RPMI 1640 media supplemented with 10% FBS and penicillin/streptomycin. SCLC cell line DMS53 was grown in Waymouth’s media supplemented with 10% FBS and penicillin/streptomycin. During cell passages, SCLC cells were dissociated with TrypLE for 10-15 minutes at 37°C. CDX-derived cells were cultured in HITES media (RPMI supplemented with 5 μg/ml insulin, 10 μg/ml transferrin, 10 nM β-oestradiol, 30 nM sodium selenite and 10 nM hydrocortisone) supplemented with 2.5% FBS and penicillin/streptomycin with the addition of 5 μM of ROCK inhibitor (Y-27632, Selleckchem). During cell passages, CDX-derived cells were dissociated with StemPro Accutase for 5-10 minutes at 37°C.

### Method details

#### RNA-seq analysis

Gene expression of NE, non-NE genes, RAC1, TIAM1, TIAM2 and MYC were obtained from the RNA-seq data of the 81 SCLC tumors published in George et al. (2015), CDX RNA-seq data published in Simpson et al. (2020), while SCLC cell line RNA-seq data were obtained from the Broad Institute. RNA-seq data were processed using the DESeq2 package (Love et al., 2014). Minimal pre-filtering retaining only samples with more than 10 reads and apeglm shrinkage estimation was applied. All bioinformatic analysis has been performed in R 4.0 (R Development Core Team, 2021).

#### NE score

For each sample the NE score has been calculated based on the method described in Zhang et al. (2018) generating a score ranging between –1 and 1 with a positive score predicting the sample being NE, and a negative score predicting a non-NE sample. The formula for the score: NE score = (correl NE – correl non-NE)/2. Correl NE (or non-NE) is the Pearson correlation between expression of the 50 Adi Gazdar signature genes in the sample of interest and expression of these genes in the NE (or non-NE) cell line group. Correlation of the NE scores with the expression of selected genes was analyzed using Spearman’s rank correlation test.

#### PCA

Principal component analysis was performed on each dataset separately. The eigenvalues were calculated based on the expression of the genes within the Adi Gazdar gene set supplemented with the expression of RAC1 and TIAM1. A more detailed description of the PCA method can be found at https://bioconductor.org/packages/release/bioc/vignettes/PCAtools/inst/doc/PCAtools.html

#### Venn diagram

A list of RhoGEFs was obtained from Müller et al. (2020). Rho GEFs were analyzed in NE (NE score > 0) and non-NE (non-NE score < 0) samples of each dataset separately. The Wilcoxon test was used to determine a significant change in expression (p < 0.05) between the NE and non-NE groupings. Significantly upregulated GEFs were then displayed in a Venn diagram highlighting their overlap between the different groups.

#### Heatmap

Heatmaps were generated for each dataset with the gplots R package (Warnes et al., 2009) using the expression of the genes within the Adi Gazdar gene set, supplemented with RAC1 and TIAM1. Unsupervised hierarchical clustering using Euclidean distances was used to construct the dendrogram.

#### *In vivo* bioluminescence assay

NTC1 or TIAM1 KO cells were transduced with retrovirus containing pMIG-Luciferase-IRES-mCherry plasmid. Infected cells were selected for mCherry expression by FACS analysis. These cells were used in mouse xenograft assay as described above. Once a week, animals were intraperitoneally injected with 150 mg/kg of D-luciferin (VivoGlow Luciferin) solution in PBS and imaged on their side on the In-Vivo Xtreme (Bruker).

#### Generation of CRISPR-Cas9-engineered cell lines

Protocols were obtained from the Zhang website https://zlab.bio/guide-design-resources. In brief, sgRNA designed to target the gene of interest was inserted into the lentiCRISPRv2-puromycin/blasticidin backbone. This plasmid was co-transfected with lentiviral packaging plasmids using Fugene® HD transfection reagent into LentiX-293T cells to generate viral particles. Lentivirus was harvested, 10 μg/μl polybrene was added and incubated with the SCLC cell lines. Virally transduced SCLC cells were selected with either puromycin or blasticidin, depending on the antibiotic resistance gene expressed by the lenti-CRISPRv2 plasmid. Gene knockout was confirmed by immunoblot analysis of whole cell lysates compared to control cell line.

#### Generation of inducible overexpression cell lines

The gene of interest to be overexpressed was PCR amplified and inserted into the pCW57 plasmid. This plasmid was co-transfected with 2^nd^ generation lentiviral packaging plasmids using Lipofectamine 2000 transfection reagent into LentiX-293T cells to generate lentivirus. SCLC cell lines were transduced with lentivirus containing doxycycline inducible gene of interest in the pCW57 plasmid as well as 10 μg/μl polybrene and selected for with neomycin/G418. SCLC cells with genome incorporated inducible genes were tested for expression of the gene of interest after 24-48 hours of doxycycline treatment.

#### Generation of TIAM1 AA mutant

The following TIAM1 human DNA sequence was mutated from GGGTCCTCAAGT ACCC ACTTCTGCTCAGGG to GGGTCCTCAAGTACCCGGCTGCGCTCAGGG using QuikChange II site directed mutagenesis (Agilent) according to the manufacturer’s instructions. The following primers were used:

Forward primer: 5′-CAGAGGGTCCTCAAGTACCCGGCTGCGCTCAGGGAGCTG TTTGCGCTGAC-3′
Reverse primer: 5′-GTCAGCGCAAACAGCTCCCTGAGCGCAGCCGGGTACTTGA GGACCCTCTG-3′

#### Generation of TIAM1 AAAA mutant

The following TIAM1 human DNA sequence were mutated from GGATCCTCAAG TACCC ACTTCTGCTCAGGG to GGATCGCCAAGTACCCAGCTGCGGCCAGGG using Q5® Site-Directed Mutagenesis (New England Biolabs) according to the manufacturer’s instructions. The following primers were used:

Forward primer: 5′-GGTACTTGGCGATCCTCTGGATGGGCTTGATGAGG-3′
Reverse primer: 5′-CAGCTGCGGCCAGGGAGCTGTTCGCCCTGACCG-3′

#### Transient siRNA silencing

Transient silencing of gene expression was achieved via siRNA transfection. siRNA oligonucleotides complexed with Lipofectamine RNAiMAX transfection reagent in Opti-MEM was prepared according to manufacturer’s guidelines. Cells were incubated with siRNA-transfection reagent complex for 72 hours, following which assays were performed as required. Control siRNAs used were either siLuciferase or siNT.

#### DNA transfection

TransIT-LT1 transfection reagent was used to transfect plasmid DNA into HEK293T cells, according to manufacturer’s protocol for protein overexpression for 48 hours followed by whole cell lysate preparation.

#### Cell counts

Cell counts were performed using the Countess II (Invitrogen). Live/dead cell differentiation was determined using 0.4% Trypan blue solution.

#### Chemical inhibitor treatments

Cells were treated with 100μM NSC-23766 or concentrations as indicated or 50 μM cisplatin overnight. 1 mg/ml of doxycycline stock solution was used to induce protein expression at dilutions specified in figure legends.

#### Cell viability assay

For IC_50_: 2 X 10ˆ3 cells in 50 μl of media were plated in black bottom 96 well plates. 50 μl of media containing varying 2-fold dilutions of NSC-23766, was added to wells containing cells and incubated at 37°C for 72 hours. At least three technical repeats were plated for each concentration of NSC-23766. 25 μl CellTiter Glo® reagent was added to each well and incubated for 30 minutes at room temperature. Luminescence (indicative of cell viability) of each well was read by plate reader.

For knockdowns: 2 X 10ˆ4 cells in 100 μl of media with siRNA and transfection reagent was added to each well of a 96 well plate. At least three technical repeats were plated for each siRNA condition. Cells in 96 well plates were incubated at 37°C for 72 hours, following which 25 μl of CellTiter Glo® reagent was added to each well, incubated for 30 minutes at room temperature and luminescence was read on a plate reader.

For knockouts: After selecting for virally transduced cells as described in Generation of CRISPR-Cas9-engineered cell lines, the cells were grown in antibiotic free media for 48 hours. Then 2 X 10ˆ4 cells in 100 μl of media was added to each well of a 96 well plate. At least three technical repeats were plated for each condition. Cells in 96 well plates were incubated at 37°C for 72 hours, following which 25 μl of CellTiter Glo® reagent was added to each well, incubated for 30 minutes at room temperature and luminescence was read on a plate reader.

#### Soft agar colony formation assay

The base layer of agar was prepared by mixing equal volumes of melted, pre-warmed (at 37°C) 1.2% agarose solution in sterilized water with 2X RPMI (supplemented with 7.5% sodium bicarbonate, 20% FBS and 2% Pen/Strep). 2 mL of the 0.6% agar-RPMI solution was added to each well of a 6 well plate and allowed to set at room temperature for 20 minutes. The top layer of agar was prepared by mixing melted, pre-warmed (at 37°C) 0.6% agarose solution in sterilized water with an equal volume of 2X RPMI. The 0.3% agar-RPMI solution was allowed to incubate at 37°C for at least 30 minutes. SCLC cells were disaggregated to produce single cell suspensions and counted. 20,000 cells were mixed well with 1 mL of 0.3% agar-RPMI solution and gently plated on top of the 2 mL of previously set 0.6% agar-RPMI layer. Three wells were prepared for each condition to obtain three technical repeats. After the top layer of agar was set at room temperature, plates were incubated at 37°C incubator for 4 weeks. Cells in each well were fed with 0.5 mL 0.3% agar-RPMI solution every 5-7 days. Colonies were stained with Indonitrotetrazolium (INT) solution (1 mg/ml). Plates were imaged and colonies counted using the “analyze particles” tool in the Fiji-ImageJ software.

#### Apoptosis assay

Cells were harvested, gently disaggregated by treatment with TrypLE and passed through filters attached to the tops of round bottom FACS tubes to obtain single cell suspensions. Each sample of cells was washed with 1 mL PBS followed by 1 mL of 1X dilution of Binding Buffer. Cells were resuspended in 100 μl of 1X Binding Buffer with 5 μl of APC fluorochrome-conjugated Annexin V and incubated for 10-15 minutes at room temperature. Cells were washed with 1X Binding Buffer and finally resuspended in 200 μl of 1X Binding Buffer. 2 μl of Propidium Iodide (PI) Staining Solution was added to each sample. Samples were analyzed by flow cytometry within 4 hours, storing the samples at 4°C in the dark in the meantime. Data were analyzed using FlowJo Software. Cells were gated to remove doublets. Recorded events of single cells were analyzed to calculate the percentage of Annexin V +ve cells. In the case of GFP +ve cell lines, cells were gated for GFP expression and then analyzed to calculate % of Annexin +ve cells.

#### Cell-cycle analysis

Cells were harvested, gently disaggregated by treatment with TrypLE and passed through filters attached to the tops of round bottom FACS tubes to obtain single cell suspensions. Each sample of cells was washed with 1 mL PBS followed by fixation with ice cold 70% ethanol for 30 minutes. Cells were washed with PBS and then resuspended in 500 μl of FxCycle PI/RNase Staining Solution. Cells were then incubated for 15 minutes before being analyzed by flow cytometry. Data were analyzed using FlowJo Software. Cells were gated to remove doublets. Subsequently, G1, S and G2/M phase gates were determined in control cells and applied to all conditions.

#### Flow cytometry analysis of BCL2 BH3 conformation change

Cells were harvested, gently disaggregated by treatment with TrypLE and passed through filters attached to the top of round bottom FACS tubes to obtain single cell suspensions. Cells were fixed with 3.7% formaldehyde for 20 minutes at room temperature. Fixed cells were washed with PBS and permeabilized with 0.1% Triton X-100 in PBS for 7 minutes. Permeabilized cells were washed with PBS and blocked with 5% BSA in PBS for 1 hour at room temperature. Cells were then immunostained with BCL2 BH3 specific antibody and Alexfluor 488 conjugated secondary antibody. BCL2 BH3 immunofluorescence was analyzed by flow cytometry using FlowJo software. Fluorescence of control DMSO or siControl treated cells (blue histogram) was compared to other treatment conditions as indicated.

#### Nuclear fractionation

Cells were collected by scraping in cold PBS or by centrifugation and washed twice with cold PBS. Each cell pellet was resuspended in 500 μl Hypotonic Buffer (20 mM Tris-HCL, pH 7.4, 10mM NaCl and 3 mM MgCl_2_) and incubated for 15 minutes on ice, after which 25 μl of 10% NP40 was added and samples vortexed at highest setting for 10 s. The homogenate was centrifuged at 720 x g at 4°C for 10 minutes. The supernatant contained the cytosolic fraction and was set aside for analysis by immunoblot, while the nuclear proteins in the pellet were extracted by resuspension in 50 μl Cell Extraction Buffer for 30 minutes on ice, vortexing at 10-minute intervals. The nuclear homogenate was centrifuged at 14,000x g at 4°C for 30 minutes. The supernatant containing the nuclear fraction was then used for immunoprecipitation or analysis by immunoblot.

#### Whole-cell lysis

Plated cells were placed on ice, scraped into media and harvested by centrifugation. Cell pellets were washed twice with cold PBS and centrifuged at 1200 x g for 5 minutes. Washed cell pellets were resuspended in IP Lysis Buffer (50mM Tris-HCl, pH 7.5, 150 mM NaCl, 1% (v/v) Triton X-100, 10% (v/v) glycerol, 2 mM EDTA, 25 mM NaF, 2 mM NaH_2_PO_4_) supplemented with 1:100 dilutions of two phosphatase inhibitor cocktails and a protease inhibitor cocktail and incubated on ice for 20 minutes. Lysed cell suspensions were centrifuged at 14,000 x g at 4°C for 10 minutes. Protein concentrations of the supernatants were measured, and equal concentrations of the different samples were prepared. Whole cell lysates were then used for immunoprecipitation or mixed with 2X sample loading buffer, denatured by heating at 70°C for 10 minutes and analyzed by immunoblotting.

#### Protein G Sepharose immunoprecipitation

Whole cell lysates prepared as described above were pre-cleared with IgG bound Protein G Sepharose beads for at least 2 hours at 4°C on a rotating wheel. Equal amounts of pre-cleared lysates were kept aside to analyze total protein levels (inputs) by immunoblotting and the remaining lysates were incubated with 1-2 μg of antibody against the protein to be immunoprecipitated or non-specific IgG as a negative control. After 1-2 hours of incubation at 4°C, BSA blocked protein G Sepharose beads were added to the lysate-antibody solutions and rotated on a wheel at 4°C for 1 hour to enable beads to bind antibody-protein complexes. Beads were collected by centrifugation at 3000 x g for 1 minute. Supernatants containing unbound proteins were discarded while beads bound to antibody-protein complexes were washed 5 times with IP lysis buffer supplemented with protease inhibitor cocktail. After the final wash, beads were resuspended in 2X sample loading buffer and heated at 70°C for 10 minutes to denature and release proteins from beads and antibody-protein complex interactions. Samples were centrifuged and the supernatants were loaded on gels for immunoblot analysis of immunoprecipitated proteins.

#### Myc-agarose immunoprecipitation

Whole cell lysates prepared as described above were pre-cleared with IgG bound Protein G Sepharose beads for at least 2 hours at 4°C on a rotating wheel. Equal aliquots of pre-cleared lysates were kept aside to analyze total protein levels (inputs) by immunoblotting and each of the remaining lysates were incubated with 40 μl of pre-washed Myc-agarose beads on a rotating wheel for 1 hour at 4°C. Supernatants containing unbound proteins were discarded while Myc-agarose-protein complexes were washed 5 times with IP lysis buffer supplemented with protease inhibitor cocktail. After the final wash, beads were resuspended in 40 μl of 2X sample loading buffer and heated at 95°C for 10 minutes to denature and release proteins from Myc-agarose and protein complex interactions. Samples were centrifuged and the supernatants were loaded on gels for immunoblot analysis of immunoprecipitated proteins.

#### RAC activity assay

RAC activity assay was performed as described in Marei et al. (2016). Briefly, cells were lysed in GST lysis buffer [25 mM Tris-HCL pH 7.2, 150 mM NaCl, 5 mM MgCl_2_, 1% Nonidet P40 (v/v), 5% glycerol (v/v), 1% protease inhibitor cocktail (v/v), 1% phosphatase inhibitor cocktails 1 and 2 (v/v) in dH2O] and subjected to biotinylated PAK-CRIB pulldown to detect levels of active RAC1. Lysates were incubated with 60 μL Strep-Tactin superflow resin together with 6 μg biotinylated PAK-CRIB purified peptide for 1 hour at 4°C. Levels of active and total RAC1 were detected by western blot analysis using a RAC1 primary antibody.

#### Immunoblotting

Proteins in whole cell lysates or immunoprecipitated protein complexes were denatured in sample loading buffer and loaded onto 10-well 4%–12% Bis-Tris or 3%–8% Tris-Acetate gels along with a protein ladder marker and separated by SDS-PAGE according to the manufacturer’s instructions. Proteins were then wet transferred from the gels onto Immobilon-P PVDF membranes using the XCell II Blot module system and protocol. Membranes containing the separated proteins were blocked in 10% milk-TBST and then incubated with primary antibodies against proteins of interest to detect protein levels in the various samples. Membranes were washed and incubated with HRP conjugated secondary antibodies (mouse and rabbit at 1:1000, and sheep secondary at 1:10000) to enable detection and measurement of the levels of primary antibodies bound to the proteins on the membrane using X-ray film and a developer machine.

#### Immunofluorescence microscopy

Cells were plated on coverslips and treated as specified. Cells on coverslips were washed with PBS and fixed onto coverslips with 3.7% formaldehyde for 20 minutes at room temperature. Fixed cells were washed with PBS and permeabilized with 0.1% Triton X-100 in PBS for 7 minutes. Permeabilized cells were washed with PBS and blocked with 5% BSA in PBS for 1 hour at room temperature. Cells on coverslips were incubated with primary antibody in 1% BSA-PBS solution overnight at 4°C followed by secondary antibodies at 1:500 dilution in 1% BSA-PBS solution for 30 minutes at room temperature. Coverslips were mounted onto glass slides using Prolong antifade mountant with DAPI and images of cells were acquired on one of two microscopes:

(1) The Decon Vision deconvolution Olympus IX83 microscope using (Blue) Lumencor LED excitation, a 60x/ 1.42 Plan Apo N or 100x/1.35 Uplan Apo oil objective lens and the (Sedat DAPI/FITC) filter set (Chroma [*89000*]). The images were collected using a R6 Qimaging CCD camera with Z optical spacing of 0.2 μm. The software used for image acquisition was Metamorph v7.10.09.119 (Molecular devices). Raw images were then deconvolved using the Huygens Pro v16.05 (SVI) software and maximum projections of these deconvolved images are shown in the Results section of this paper.
(2) The laser scanning confocal microscope, Zeiss LSM880 Upright Airyscan with argon laser 458, 488, 514 (Lasos, Jena, Germany), Diode 405-30 (Lasos), DPSS 561-10 (Lasos) HeNe 633 nm (Lasos), a Plan-Apochromat 63 x/1.4 Oil DIC M27 (Zeiss) objective lens. Equipment control, image acquisition and processing of raw images were performed by Zen Black (Zeiss) software and maximum projections of the images are shown in the results.

All processed images were analyzed in Fiji-ImageJ software (Schindelin et al., 2012), where images in a given figure panel, stained with the same antibody were set to identical minimum and maximum brightness for comparison of protein localization and intensity.

### Quantification and statistical analysis

#### Nuclear/cytoplasmic ratio quantification

Nuclear to cytoplasmic ratio of Nur77 immunofluorescence was determined by the “Intensity Ratio Nuclei Cytoplasm” tool in the ImageJ software. The tool utilized DAPI stained images to create masks around the nucleus and separately measured the average fluorescence intensity of the FITC channel (Nur77) in the nucleus and immediately outside the nucleus (cytoplasmic).

#### Statistical analysis

RNA-seq datasets were analyzed using R version 4.0. To determine correlation between two variables, Spearman’s rank correlation was used. To determine if genes were significantly differentially expressed between groups, the Wilcoxon rank sum test was used. For all other datasets the statistical significance of differences between groups was analyzed using Prism (GraphPad) software. The statistical test used and the definition of n are indicated in figure legends. Comparisons of groups only to the control were performed using unpaired two-tailed Student’s t tests. For multiple comparisons, one-way or two-way ANOVA was performed depending on the number of independent variables. In both cases Sidak’s multiple comparisons test was used to determine statistical significance. Results were considered statistically significant when p < 0.05. All data are presented as mean -/+ SEM. In all figures: ^∗∗∗∗^p < 0.0001, ^∗∗∗^p < 0.001, ^∗∗^p < 0.01, ^∗^p < 0.05, ns: non-significant.

## Data Availability

• Original western blot images, plate reader measurements, flow cytometry measurements, and original microscopy images reported in this paper will be shared by the lead contact upon request. • All original code has been deposited at Zenodo and is publicly available as of the date of publication. The DOI is listed in the Key resources table. • Any additional information required to reanalyze the data reported in this paper is available from the lead contact upon request.

## Supplemental information

Document S1. Figures S1–S6 and Table S1

Document S2. Article plus supplemental information
