# Supplementary material for: TIAM1-RAC1 promote small-cell lung cancer cell survival through antagonizing Nur77-induced BCL2 conformational change
Source: Cell Rep. 2021 Nov 9;37(6):109979. doi: 10.1016/j.celrep.2021.109979 (PMC8595642; doi:10.1016/j.celrep.2021.109979)
Supplement: Document S1. Figures S1–S6 and Table S1 [file mmc1.pdf]

Cell Reports, Volume *37*

## Supplemental information

# TIAM1-RAC1 promote small-cell lung cancer cell survival through antagonizing Nur77-induced BCL2 conformational change

**Aishwarya Payapilly, Ryan Guilbert, Tine Descamps, Gavin White, Peter Magee, Cong Zhou, Alastair Kerr, Kathryn L. Simpson, Fiona Blackhall, Caroline Dive, and Angeliki Malliri**

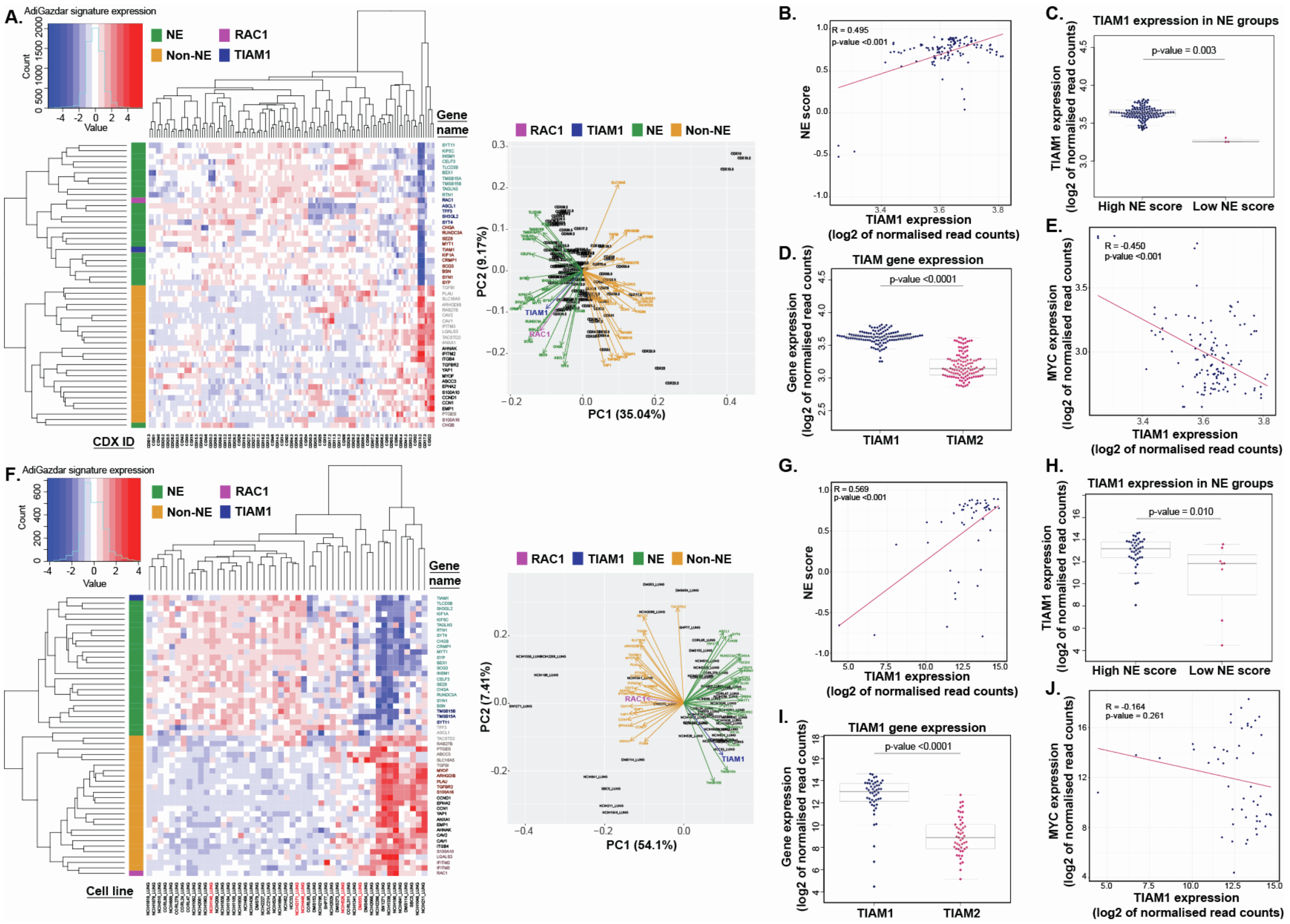


**Figure S1. TIAM1-RAC1 signaling is associated with NE SCLC. Related to Figure 1.**

**(A)** Hierarchical clustering based on NE (green), non-NE (yellow), TIAM1 (blue) and RAC1 (pink) gene expression in SCLC CDX tumors represented by a heat map and PCA plot. **(B)** Spearman correlation between 50 gene NE scores calculated for each CDX tumor and TIAM1 gene expression. p-value <0.001 (Spearman correlation test). **(C)** Comparison of TIAM1 gene expression in either high NE score or low NE Score CDX tumors. Box plots represent interquartile range with median TIAM1 gene expression. p-value =0.003 (Wilcoxon rank sum test). **(D)** Comparison of TIAM1 vs TIAM2 gene expression in SCLC patient tumors. Box plots represent interquartile range with median gene expression. p-value <0.0001 (Wilcoxon rank sum test). **(E)** Spearman correlation between MYC and TIAM1 gene expression in CDX tumors. p-value <0.001 (Spearman correlation test). **(F)** Same as (A) but for SCLC cell lines. **(G)** Same as (B) but for SCLC cell lines. p-value <0.001. **(H)** Same as (C) but for SCLC cell lines. p-value =0.01. **(I)** Same as (D) but for SCLC cell lines. p-value <0.0001. **(J)** Same as (E) but for SCLC cell lines. p-value =0.261.

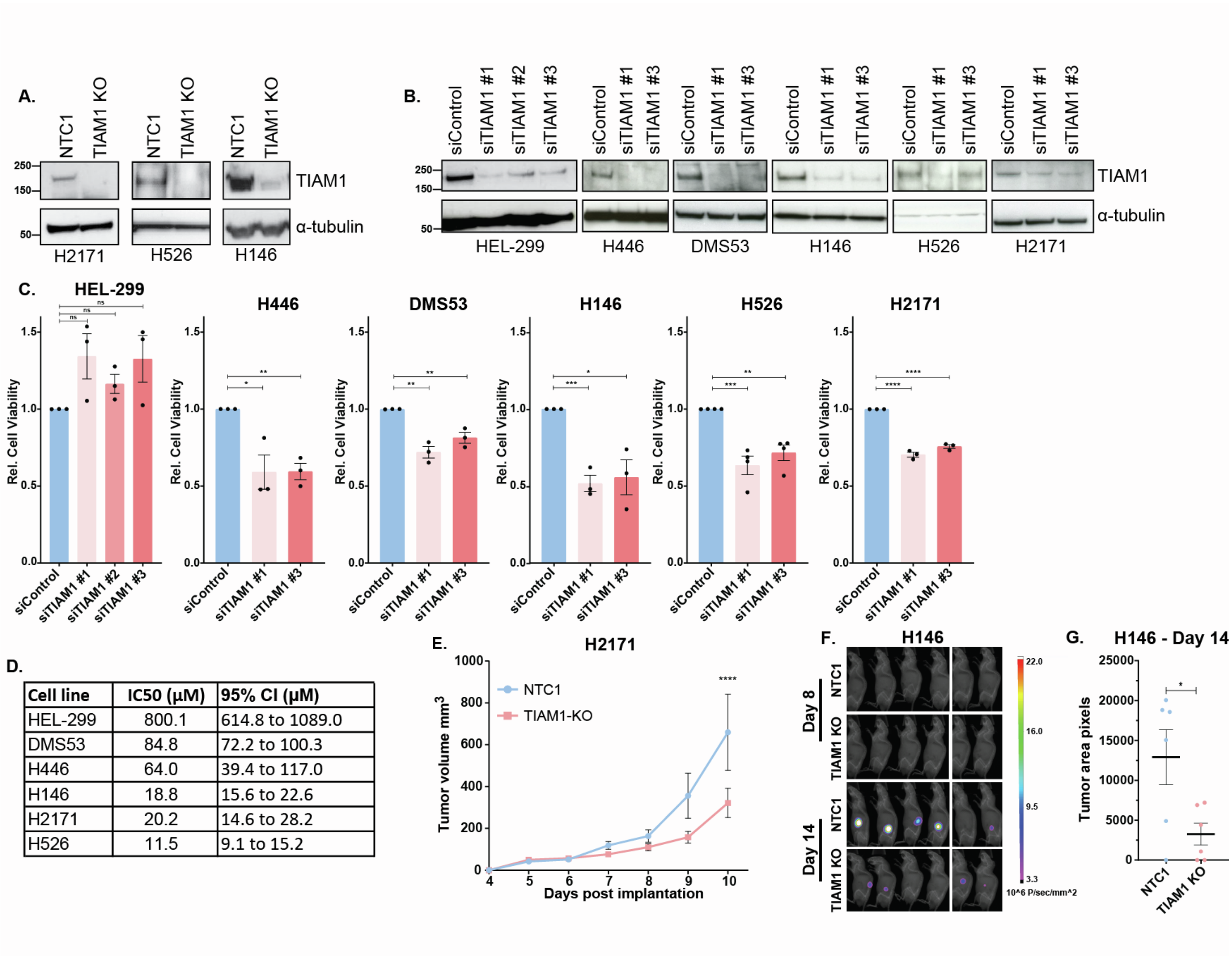


| Cell line | IC50 (µM) | 95% CI (µM) |
|---|---|---|
| HEL-299 | 800.1 | 614.8 to 1089.0 |
| DMS53 | 84.8 | 72.2 to 100.3 |
| H446 | 64.0 | 39.4 to 117.0 |
| H146 | 18.8 | 15.6 to 22.6 |
| H2171 | 20.2 | 14.6 to 28.2 |
| H526 | 11.5 | 9.1 to 15.2 |

**Figure S2. TIAM1 promotes SCLC viability and tumorigenic potential of SCLC cells. Related to Figure 2.**

**(A)** TIAM1 expression in indicated SCLC cell lines following TIAM1 knockout (KO). Western blots representative of 3 independent experiments. **(B)** Immunoblots showing TIAM1 knockdown, with at least two independent siRNAs, in HEL-299 and SCLC cell lines as indicated. Western blots representative of 3 independent experiments. **(C)** Quantification of fold change in cell viability of siRNA treated cell lines of (B). Errors bars= -/+ SEM from n=3 independent experiments. *p=0.0210 for siTIAM1#1, **p=0.0016 for siTIAM1#3 in H446; **p=0.0018 for siTIAM1#1, **p=0.0064 for siTIAM1#3 in DMS53; ***p=0.0008 for siTIAM1#1, *p=0.0173 for siTIAM1#3 in H146; ***p=0.0009 for siTIAM1#1, **p=0.0013 for siTIAM1#3 in H526; ****p<0.0001 for siTIAM1#1, ****p<0.0001 for siTIAM1#3 in H2171, ns=non-significant. (All significance tests were unpaired t-test, two-tailed.). **(D)** Table shows IC50 values, and 95% CI values obtained for the NSC-23766 dose responses obtained in Figure 2C. **(E)** Volume of subcutaneous tumors of control (NTC1) or TIAM1 KO H2171 cells measured over time, n=5 mice per condition. Error bars= -/+ SEM, ****p<0.0001 (two-way ANOVA,

Sidak's multiple comparison test). **(F)** Representative bioluminescent images of mice subcutaneously injected with either H146-NTC1 or H146-TIAM1 KO bioluminescent cells on indicated days following implantation. **(G)** Quantitation of pixel area for tumors shown in (F). Error bars= -/+ SEM from n=6 mice. *p=0.0262 (unpaired t-test, two-tailed).

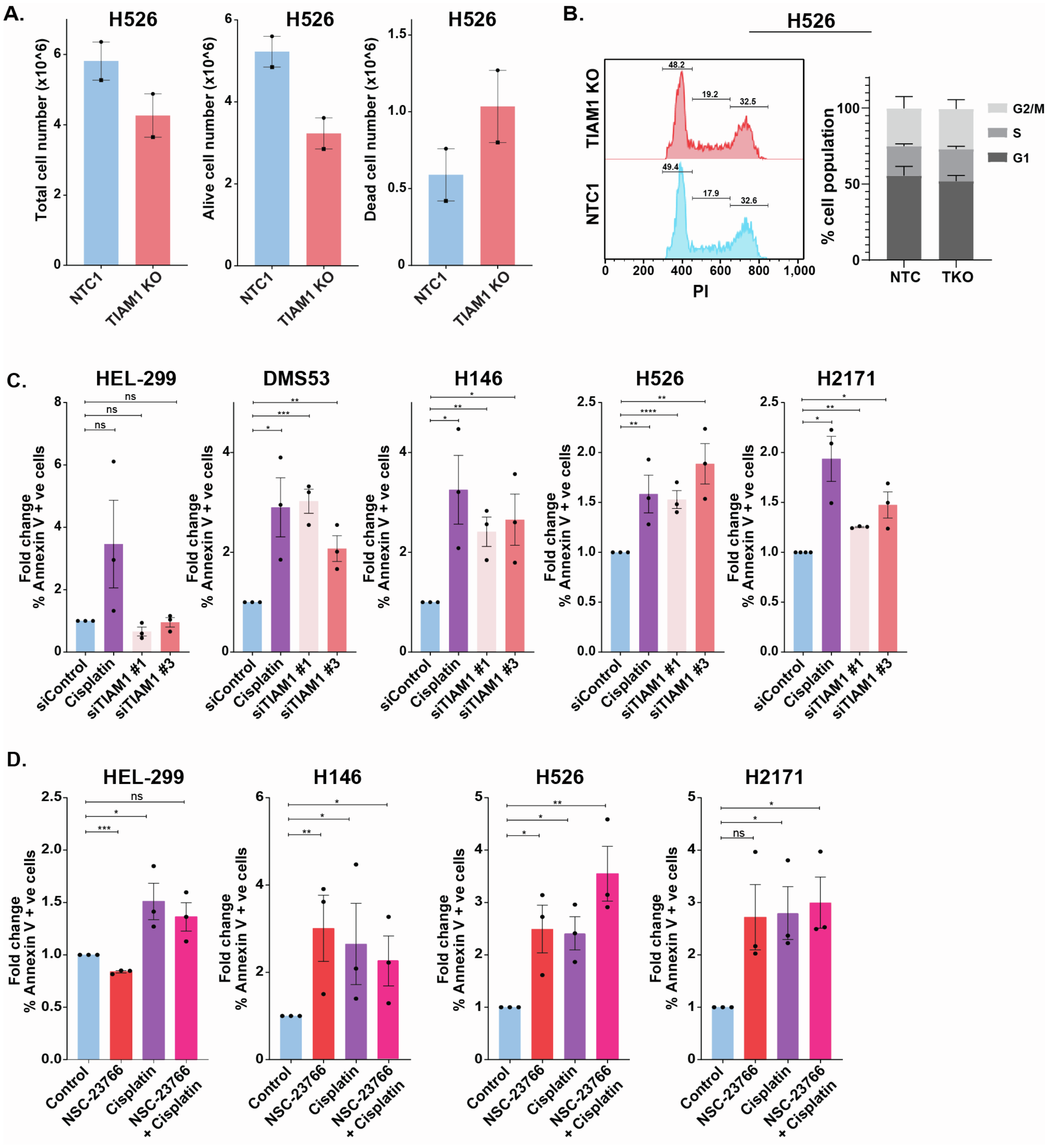


## Figure S3. Inhibition of TIAM1-RAC1 induces BAX/BAK mediated apoptosis in SCLC cells. Related to Figure 3.

**(A)** Counts of TIAM1 KO vs control (NTC1) H526 cells. TIAM1 was depleted by lenti-CRISPR and cells were selected with puromycin. Equal number of control and KO cells were plated, and 72 hours later cell counts were taken. Trypan blue solution was used for live/dead cell differentiation. Error bars= -/+ SEM from n=2 independent experiments. **(B)** Flow cytometric analysis of cell cycle distribution of H526 TIAM1 KO vs control (NTC1) H526 cells. TIAM1 was depleted by lenti-CRISPR and cells were selected with puromycin. Equal number of control and KO cells were plated, and 72

hours later cell cycle analysis using PI was performed. Quantification of n=2 independent experiments shown on the right, error bars= -/+ SEM. **(C)** Fold change in % Annexin V +ve cells for cells treated with Cisplatin or siRNA against TIAM1, compared to control siRNA treated cells in HEL-299 and SCLC cell lines. Error bars= -/+ SEM from n=3 independent experiments. *p=0.0121 for Cisplatin, ***p=0.0002 for siTIAM1#1, **p=0.0041 for siTIAM1#3 in DMS53; *p=0.0307 for Cisplatin, **p=0.0087 for siTIAM1#1, *p=0.0322 for siTIAM1#3 in H146; **p=0.0042 for Cisplatin, ****p<0.0001 for siTIAM1#1, **p=0.0076 for siTIAM1#3 in H526; *p=0.0364 for Cisplatin, **p=0.0041 for siTIAM1#1, *p=0.0119 for siTIAM1#3 in H2171; ns=non-significant. (All significance tests were unpaired t-test, two-tailed.) **(D)** Fold change in % Annexin V +ve cells for cells treated with Cisplatin, NSC-23766 or both, compared to control DMSO treated cells in HEL-299 and SCLC cell lines. Error bars= -/+ SEM from n=3 independent experiments. ***p=0.0001 for NSC-23766, *p=0.0427 for Cisplatin in Hel-299; **p=0.0049 for NSC-23766, *p=0.0304 for Cisplatin, *p=0.0119 for Cisplatin+NSC-23766 in H146; *p=0.0306 for NSC-23766, *p=0.0111 for Cisplatin, **p=0.0083 for Cisplatin+NSC-23766 in H526; *p=0.0234 for Cisplatin, *p=0.0149 for Cisplatin+NSC-23766 in H2171; ns=non-significant. (All significance tests were unpaired t-test, two-tailed.)

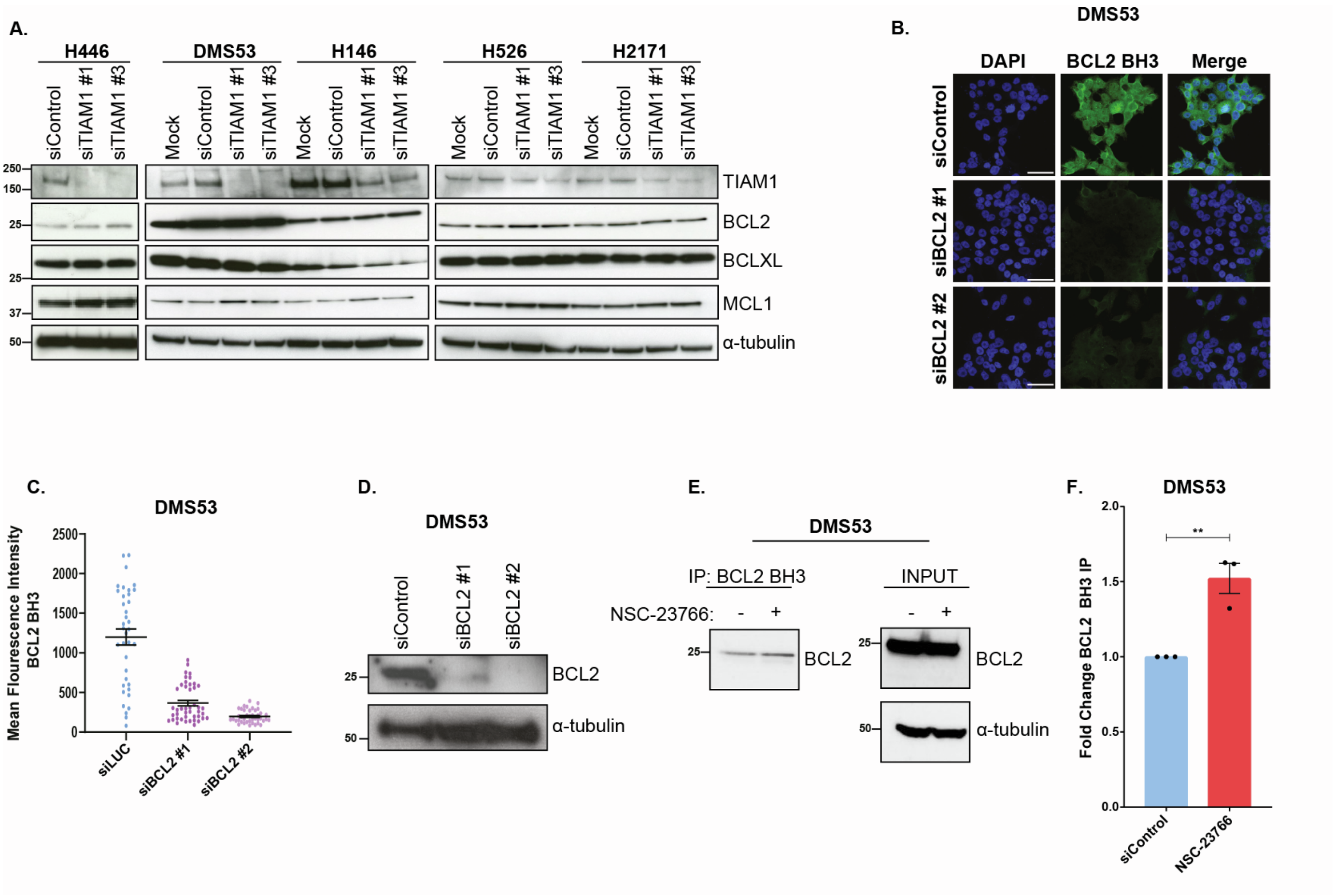


**Figure S4. Inhibition of TIAM1-RAC1 induces BCL2 conformational change in SCLC cells. Related to Figure 4.**

**(A)** Immunoblots showing expression of TIAM1, BCL2, BCLXL and MCL1 in control siRNA and TIAM1 siRNA-treated SCLC cancer cell lines. **(B)** Representative images of siControl or siBCL2 treated DMS53 cells stained with the BCL2 BH3 domain specific antibody. Scale bar: 50 μm. **(C)** Quantification of mean intensity of BCL2 BH3 antibody staining of (B). Each data point represents one cell with n>35 cells for each condition. **(D)** Western blot showing BCL2 levels from control siRNA treated cells or when treated with two independent siRNAs against BCL2. **(E)** Representative western blot of BH3 domain exposed BCL2 immunoprecipitated from control or NSC-23766 treated DMS53 cells. **(F)** Quantification of (E). Error bars= -/+ SEM of n=3 independent experiments; **p=0.0032 (unpaired t-test, two-tailed).

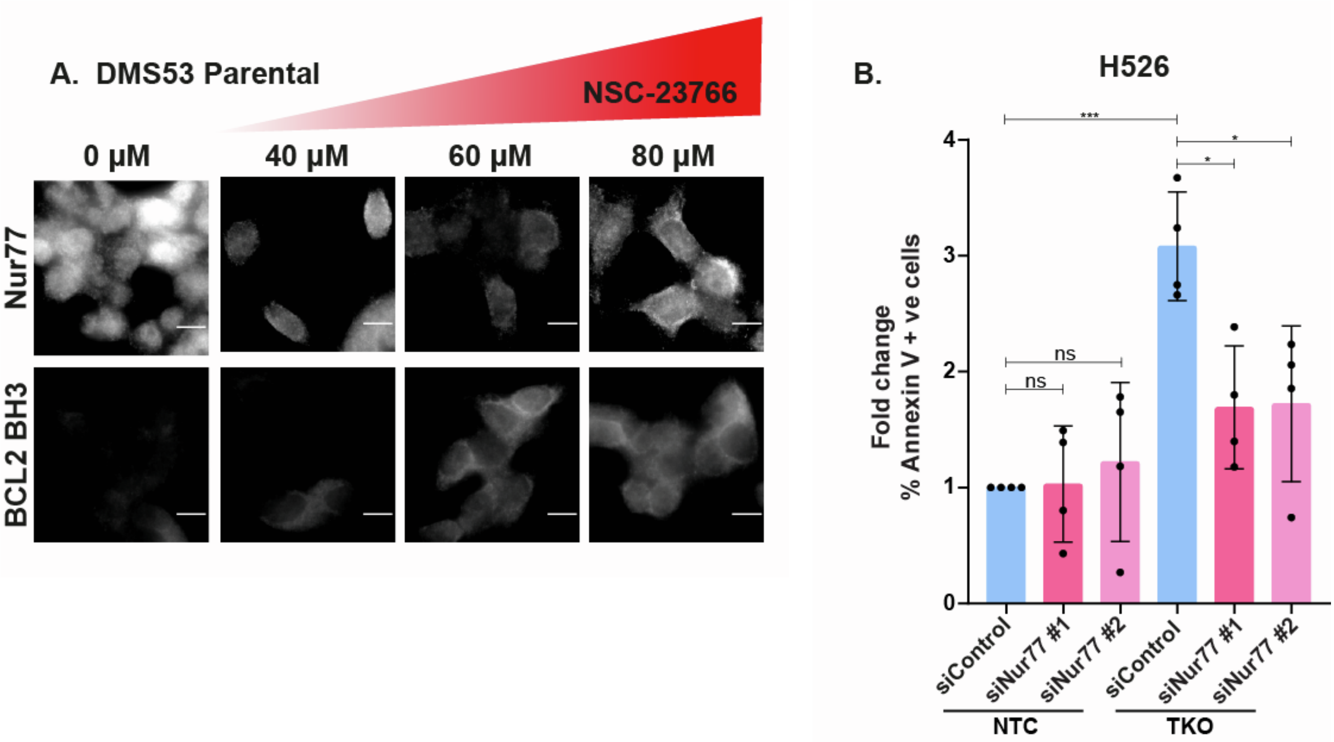


**Figure S5. Nur77 mediates BCL2 BH3 domain exposure and apoptosis upon inhibition of TIAM1-RAC1 signaling. Related to Figure 5.**

**(A)** Representative images of Nur77 localization or BCL2 BH3 staining following treatment of cells with DMSO or increasing concentrations (40 μM, 60 μM, 80 μM) of NSC-23766. Scale bars: 10 μm. **(B)** Fold change of Annexin V +ve control (NTC) or TIAM1 KO (TKO) H526 cells treated with either control siRNA or two independent siRNAs against Nur77. Error bars= -/+ SEM for n=3 independent experiments. ***p=0.0004 for siControl NTC vs siControl TKO cells, *p=0.0231 for siControl TKO vs siNur77 #1 TKO cells, *p=0.0280 for siControl TKO vs siNur77 #2 TKO cells. ns=non-significant. (For all comparisons two-way ANOVA, Sidak's multiple comparisons test.)

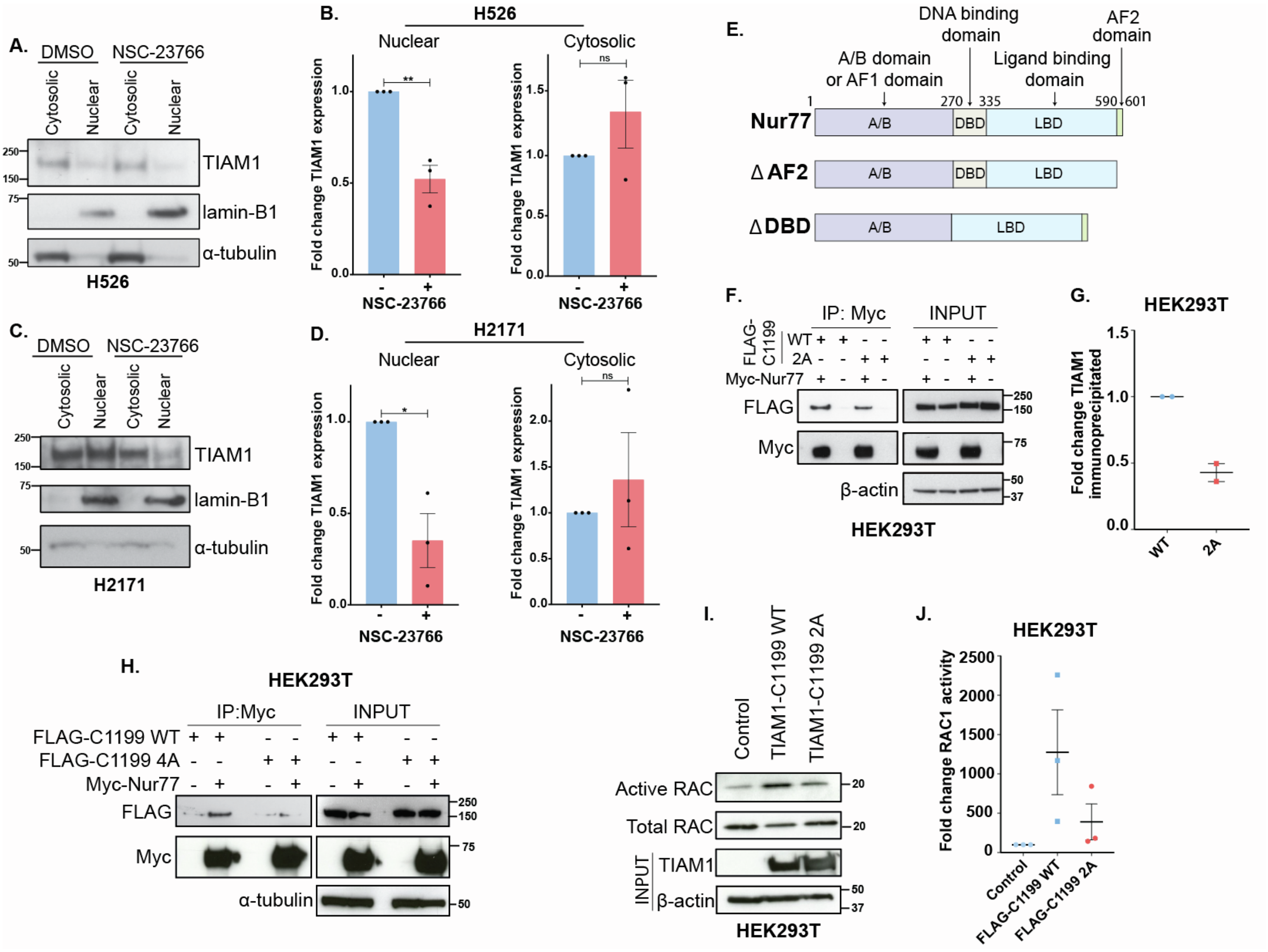


**Figure S6. Nuclear TIAM1 levels in SCLC cells are regulated by RAC. Molecular characterization of the interaction between Nur77 and TIAM1. Related to Figure 6.**

**(A)** Immunoblot showing TIAM1 levels in the nucleus and cytoplasm of H526 cells treated with either 50μM NSC-23766 or DMSO overnight. **(B)** Quantification of (A). Graphs show relative quantification of TIAM1 levels in the nucleus and cytosol of H526 cells treated with 50μM NSC-23766 or DMSO normalized to nuclear or cytosolic loading control respectively. Error bars= -/+ SEM from n=3 independent experiments. For nuclear comparison, **p=0.0032; for cytosolic comparison, ns=non-significant (unpaired t-test, two-tailed). **(C)** Same as (A) but for H2171. **(D)** Same as (B) but for H2171. For nuclear comparison *p=0.0144, for cytosolic comparison, ns=non-significant (unpaired t-test, two-tailed). **(E)** Schematic of the protein domains of Nur77 showing the N-terminal A/B domain or activation function 1 (AF1) domain, DNA binding domain (DBD), ligand binding domain (LBD) and C-terminal AF2 domain. **(F)** Representative immunoblot showing the interaction of wildtype (WT) or 2A mutant C1199-TIAM1 with Myc-Nur77 following their transfection in HEK293T cells and

immunoprecipitation with anti-Myc. **(G)** Quantification of (F). Error bars= -/+ SEM from 2 independent experiments. **(H)** Representative immunoblot showing the interaction of wildtype (WT) or 4A mutant of C1199-TIAM1 with Myc-Nur77 following their transfection in HEK293T cells and immunoprecipitation with anti-Myc. **(I)** Representative immunoblot of RAC1 activation by either control, TIAM1-C1199 or TIAM1-2A mutant in HEK293 cells. **(J)** Quantification of (I). Error bars= -/+ SEM from n=3 independent experiments.

**Table S1: List of oligonucleotides used in this study. Related to STAR Methods.**

| Oligonucleotides | SOURCE | IDENTIFIER |
|---|---|---|
| sgRNA NTC1<br>5’-GCTGAAAAAGGAAGGAGTTGA-3’ | (Joung et al., 2017) | N/A |
| sgRNA TIAM1<br>5’-GCTCATCGCTGTGCGCCGAGC-3’ | (Diamantopoulou et al., 2017) | N/A |
| sgRNA BAX<br>5′-AGTAGAAAAGGGCGACAACC-3’(−) | (Lopez et al., 2016) | N/A |
| sgRNA BAK<br>5′-GCCATGCTGGTAGACGTGTA-3′(−) | (Lopez et al., 2016) | N/A |
| sgRNA Nur77 #1<br>5’-GCCCATATTGGGCTTGGATAC-3’ | This study | N/A |
| sgRNA Nur77 #2<br>5’-GTCGCCCAGCCAGACTTACGA-3’ | This study | N/A |
| siControl/siLuciferase<br>5’-CGUACGCGGAAUACUUCGA-3’ | This study | N/A |
| siControl/siNT<br>Silencer™ Select Negative Control No.1 siRNA | ThermoFischer Scientific UK | Cat# 4390843 |
| siTIAM1 #1<br>5’-GAGGUUGCAGAUCUGAGCA-3’ | (Vaughan et al., 2015) | N/A |
| siTIAM1 #2<br>5’-GAGACUCCUCCGUACAGUAAUUAUATT-3′ | This study | N/A |
| siTIAM1 #3<br>5’-CAGCACAACCCUGACUGCGACAUUUTT-3′ | This study | N/A |
| siNur77 #1<br>5′-CCGCUUUGGAAAGGAAGAUTT-3′ | This study | N/A |
| siNur77 #2<br>5′-CAGUCCAGCCAUGCUCCUCUU-3′ | This study | N/A |
| siBCL2 #1<br>Silencer™ 202464 | ThermoFischer Scientific UK | AM16708 |
| siBCL2 #2<br>Silencer™ 214533 | ThermoFischer Scientific UK | AM16708 |
